# *SNTA1*-deficient human cardiomyocytes demonstrate hypertrophic phenotype and calcium handling disorder

**DOI:** 10.1186/s13287-022-02955-4

**Published:** 2022-06-30

**Authors:** Tao Dong, Yan Zhao, Hai-Feng Jin, Lei Shen, Yan Lin, Long-Long Si, Li Chen, Ji-Cheng Liu

**Affiliations:** 1 Basic Medicine School, Qiqihar Medical University, 333 Bukui Street, Qiqihar, 161006 Heilongjiang China; 2 College of Life Science and Agroforestry, Qiqihar University, Qiqihar, 161006 Heilongjiang China; 3 Qiqihar Institute of Medical and Pharmaceutical Sciences, Qiqihar Medical University, 333 Bukui Street, Qiqihar, 161006 Heilongjiang China; 4 CAS Key Laboratory of Quantitative Engineering Biology, Shenzhen Institute of Synthetic Biology, Shenzhen Institute of Advanced Technology, Chinese Academy of Sciences, Shenzhen, 518055 China

**Keywords:** Human embryonic stem cell, *SNTA1*-deficient cardiomyocyte, CRISPR-Cas9, Calcium homeostasis

## Abstract

**Background:**

α-1-syntrophin (SNTA1), a protein encoded by *SNTA1*, is highly expressed in human cardiomyocytes. Mutations in *SNTA1* are associated with arrhythmia and cardiomyopathy. Previous research on SNTA1 has been based on non-human cardiomyocytes. This study was designed to identify the phenotype of *SNTA1*-deficiency using human cardiomyocytes.

**Methods:**

*SNTA1* was knocked out in the H9 embryonic stem cell line using the CRISPR-Cas9 system. H9SNTA1KO cells were then induced to differentiate into cardiomyocytes using small molecule inhibitors. The phenotypic discrepancies associated with *SNTA1*-deficient cardiomyocytes were investigated.

**Results:**

*SNTA1* was truncated at the 149th amino acid position of PH1 domain by a stop codon (TGA) using the CRISPR-Cas9 system. *SNTA1*-deficiency did not affect the pluripotency of H9SNTA1KO, and they retain their in vitro ability to differentiate into cardiomyocytes. However, H9SNTA1KO derived cardiomyocytes exhibited hypertrophic phenotype, lower cardiac contractility, weak calcium transient intensity, and lower level of calcium in the sarcoplasmic reticulum. Early treatment of *SNTA1*-deficient cardiomyocytes with ranolazine improved the calcium transient intensity and cardiac contractility.

**Conclusion:**

*SNTA1*-deficient cardiomyocytes can be used to research the etiology, pathogenesis, and potential therapies for myocardial diseases. The *SNTA1-*deficient cardiomyocyte model suggests that the maintenance of cardiac calcium homeostasis is a key target in the treatment of myocardial-related diseases.

**Supplementary Information:**

The online version contains supplementary material available at 10.1186/s13287-022-02955-4.

## Introduction

SNTA1 is an important signaling scaffold protein between the extracellular matrix and the intracellular cytoskeleton by connecting with the dystrophin-associated protein complex (DAPC) [1]. The SNTA1 binds the motif of PDZ domain of Nav1.5. Nav1.5 is an important type of cardiac voltage-gated sodium channel. SNTA1 plays a critical auxiliary role in the correct subcellular localization, expression, and function of Nav1.5. SNTA1 is involved in the regulation of membrane volume on Kir2.1and Kir2.2 channels [2]. In Snta1 knockout mice, left ventricular posterior wall thickening and abnormal myocardial performance index have been reported, indicating myocardial hypertrophy in the knockout mice [3]. The mutation of *SNTA1* can cause long QT syndrome [4, 5], Brugada syndrome [6], sudden infant death syndrome [7], and other heart-related disease. The physiological function of SNTA1 is closely related to cardiomyocytes. In addition, the PH1 domain and PDZ domain of SNTA1 have been reported to bind calmodulin [8] in a calcium-independent manner and a calcium-dependent manner [9]. Calmodulin transduces calcium signals by binding to calcium and further interacting with its downstream target proteins in the cardiomyocytes [10]. These interactions indicate a potential role of SNTA1 in cardiomyocyte calcium handling.

Calcium handling is essential for function of cardiomyocytes. Calcium is a critical intracellular signaling molecule, which mediates various biological processes, including excitation–contraction coupling (EC), enzyme activity, gene transcription, and cell death [11, 12]. As an EC factor, calcium is necessary for heart contraction. The transport and storage of calcium in cardiomyocytes received special attention because abnormal calcium handling plays a key role in the pathogenesis of cardiomyopathy and arrhythmia [13, 14]. The calcium voltage-gated channel subunit alpha1 C (CACNA1C) in T-tubules induced the extracellular calcium influx into the cell (calcium sparks formation) by the depolarized cell membrane. The calcium sparks activated the RYR2 channel on the sarcoplasmic reticulum (SR), and then the calcium in the SR is released through the RYR2 into the cytoplasm. Elevated free calcium in the cytoplasm causes myofilament contraction. Three proteins help in maintaining the function of the T-tubule in the calcium-induced calcium release (CICR) process [15]. The first protein is junctophilin 2 (JPH2), which is the primary structural protein in cardiomyocytes between the T-tubules and the SR. It drags the T-tubules closer to SR to form the junctional membrane complex, facilitating CICR [16–19]. The second protein is caveolin 3 (CAV3), a member of the caveolin protein family that contributes to the formation of caveolae and provides microdomains for a variety of functional proteins in the T-tubules [16, 20, 21]. The third protein is bridging integrator 1 (BIN1), which interacts between the BAR domain and phospholipid acid in the cell membrane to deform the membrane bilayer. BIN1 is not only involved in the formation of the T-tubules but also transports the CACNA1C to the cell membrane and helps to maintain the CICR function [22]. After contraction, SERCA2a is a macromolecular complex on the SR that reuptakes about 70% of cytosolic calcium back into the SR, facilitating cardiac relaxation, about 28% of cytosolic calcium is extruded by the NCX (sodium-calcium exchanger), and about 1% of each cytosolic calcium to be removed by the sarcolemma Ca^2+^-ATPase and mitochondrial Ca^2+^ uniporter.

Currently, the results of research on SNTA1 are from non-human cells, such as CHO cells, H9C2 cells, *Xenopus* oocytes. Patients’ induced pluripotent stem cells (iPSCs) can be used to generate a large number of patient cardiomyocytes. Patients’ cardiomyocytes were studied for abnormal phenotypes, however, there are a lot of differences between patients and normal donors in terms of the genetic background. Further study on patients' iPSCs was limited by different genetic backgrounds. With the widespread applications of gene-editing technologies in eukaryotic cells [23–25], the formation of genetically edited human embryonic stem cells can help overcome the barrier of genetic diversity [26, 27].Using the CRISPR-Cas9 system we established the H9SNTA1KO from the H9 embryonic stem cells, and then SNTA1-deficient cardiomyocytes were inducted from the H9SNTA1KO. The phenotype of SNTA1-deficient cardiomyocytes was investigated. This research provides an example of using human cells to study the phenotype of cardiomyocytes caused by the *SNTA1* knockout, while also demonstrating how human cardiomyocytes can be used to investigate the process of the gene knockout pathogenesis.

## Methods

### Embryonic stem cell culture and the design for *SNTA1* knockout

The WiCell Research Institute Inc. provided the H9 embryonic stem cell (Additional file 1). The H9 cells were cultured in an E8 medium and digested with 0.5 mM EDTA when they reached 80% confluence. Zhang Lab web tools were used to design single guide RNA (sgRNA) targeting *SNTA1* using the CRISPR-Cas9 system. The sgRNA targeting site was used to select public exons close to the start codon: we selected exon 2 design sgRNA (5′-attggcaggacag-3′) and confirmed deletion by western blotting. H9SNTA1KO cell line was established.

### Cardiac differentiation

The H9 embryonic stem cell and H9SNTA1KO were induced to differentiate into cardiomyocytes using CardioEasy kit containing small molecule inhibitors (Cellapy, China).

### RNA-sequencing (RNA-seq) analysis and Quantitative Real-time PCR (qRT-PCR)

After RNA was extracted from SNTA1-deficient cardiomyocytes (KO-cardiomyocytes) and wild type (WT)-cardiomyocytes, RNA-seq was analyzed by BGI Tech. Solutions Co., Ltd. (Liuhe, China). Total cellular RNA was extracted with TRIzol (Invitrogen, USA) and treated with DNase I (Beyotime, China) for approximately 30 min at 37 °C to eliminate DNA contamination. RNA was reverse transcribed using the Prime-Script TM reverse transcription system (TaKaRa, Japan). Relative gene expression levels were examined by qRT-PCR using the iCycler iQ5 (Bio-Rad, USA) with TB Green™Premix Ex Taq™II (Takara, Japan). The relative quantification was calculated according to the ▵CT method. The primer sequences used for qRT-PCR are listed in Additional file 2: Table S1.

### Flow cytometry

The cells were digested with 0.5 mM EDTA to prepare single-cell suspensions and then incubated with the antibody for 30 min in phosphate buffered saline (PBS) at room temperature (RT). The samples were detected by Flow cytometer (Beckman, EPICS XL) and the results were analyzed using the Flow Jo VX software.

### Immunofluorescent staining

The cells were fixed in 4% paraformaldehyde for 30 min, washed three times in PBS, permeabilized with 0.3% Triton X-100 for 10 min at RT, and then blocked in 3% BSA for 30 min at RT. Then the cells were incubated with the primary antibody for 24 h at 4 °C, washed three times in PBS, and then the cells were incubated with the secondary antibody and DAPI (100 nM) for 1 h at RT. The cells were subsequently washed again three times in PBS and imaged with the confocal microscopy (Leica, TCS5 SP5). The primary and secondary antibodies used for immunofluorescent staining and western blotting are listed in Additional file 2: Table S2.

### Calcium transient assay and caffeine-evoked calcium release test

The cardiomyocytes were plated on a 35 mm confocal dish loaded with 4 μM Fluo-4 AM (Yeasen, China) and incubated at 37 °C for 20 min in PBS (Servicebio, China) containing 0.04% Pluronic F-127 (Yeasen, China). PBS was changed to the Cardiomyocytes Maintenance Medium (Cellapy, China). Loaded samples were transferred under a TCS‐SP5‐RS confocal microscope (Leica, Germany). Laser emission at 488 nm was used for stimulation and emitted fluorescence at 530 nm was acquired. Samples were then stimulated with freshly prepared solution of caffeine (20 mM) and emitted fluorescence acquired to record transient alteration in cytosolic calcium levels.

### Contractility measurement

According to the previously reported protocols, the contractility of cardiac myocytes was measured [28, 29].

### Statistical methods

The data of measurement and count were all presented as mean ± standard deviation. The rate was compared by the Fisher’s Exact test, and the difference between two groups was analyzed by one-tail or two-tail Student’s *t* test. Three or more groups of data were analyzed using single-factor or two-factor analysis of variance, followed by the Tukey multiple comparison test. The confidence interval was 95%, *P < 0.05, **P < 0.01, ***P < 0.001, ****P < 0.0001, representing four levels of statistical significance.

## Results

### Establishment of homozygous *SNTA1*-deficient hESCs (H9SNTA1KO)

**Fig. 1:**
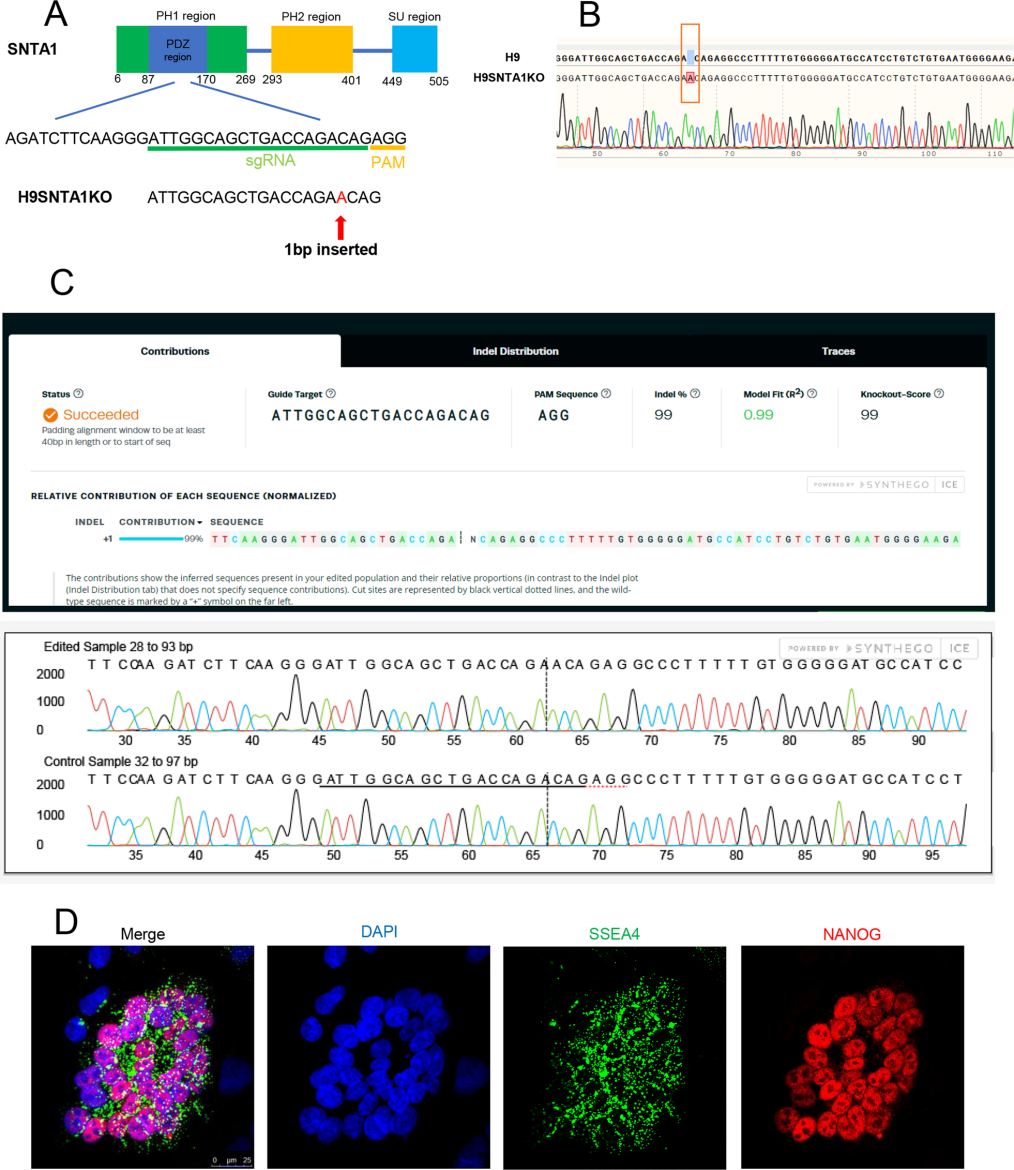
Establishment of homozygous *SNTA1*-deficient hESCs. **A** Schematic of the sgRNA designed for the PH1 region in *SNTA1* demonstrates that one adenine nucleotide is inserted into *SNTA1* before the PAM sequence. **B** The DNA molecules of H9 and H9SNTA1KO cells were detected by DNA sequencing. The DNA was extracted and amplified by PCR. The result showed one adenine nucleotide was inserted into the *SNTA1* before the PAM sequence in the H9SNTA1KO genome. **C** Utilizing tools from the Synthego website to assess the gene-editing ratio. The upper graph offered the abstract of the indel ratio with the results showing a knockout score of 99%. The relative contribution of the sequence is a nucleotide was inserted before the PAM sequence. The graph below offered the analysis of the sequence using the Synthego tools. The red dotted line is under the PAM sequence in the control sample diagram. The vertical dotted line demonstrated one adenine inserted in the edited sample diagram. The homozygous SNTA1 knockout hESCs were established. **D** Immunofluorescence staining for pluripotency was performed. Both SSEA4 and NANOG were positive in H9SNTA1KO. *SNTA1*-knockout did not influence the pluripotency of hESCs

We selected the second exon of *SNTA1,* corresponding to the pleckstrin homology 1 (PH1) domain, as the target site of sgRNA (Fig. 1A). After gene-editing, an adenine nucleotide was inserted before the protospacer adjacent motif (PAM) region, according to DNA sequencing result (Fig. 1B). A stop codon (TGA) appeared at the 149th amino acid position in the SNTA1 and terminated the SNTA1 protein prematurely in the PH1 domain. The rate of *SNTA1* knockout was evaluated using the Synthego analysis sequencing result (Fig. 1C). On H9SNTA1KO, immunofluorescence staining for pluripotency was performed. With H9SNTA1KO, both SSEA4 and NANOG were positive (Fig. 1D). The expression of pluripotent markers *SOX2*, *DPPA4*, *OCT-4,* and *NANOG* in H9SNTA1KO was similar to WT using qRT-PCR analysis (Additional file 2: Fig. 1A). The karyotype analysis of H9SNTA1KO was normal (46, XX) (Additional file 2: Fig. 1B). Furthermore, western blotting confirmed that H9SNTA1KO is SNTA1 deficient (Additional file 2: Fig. 1C) [27].

### H9SNTA1KO differentiated into cardiomyocytes

**Fig. 2:**
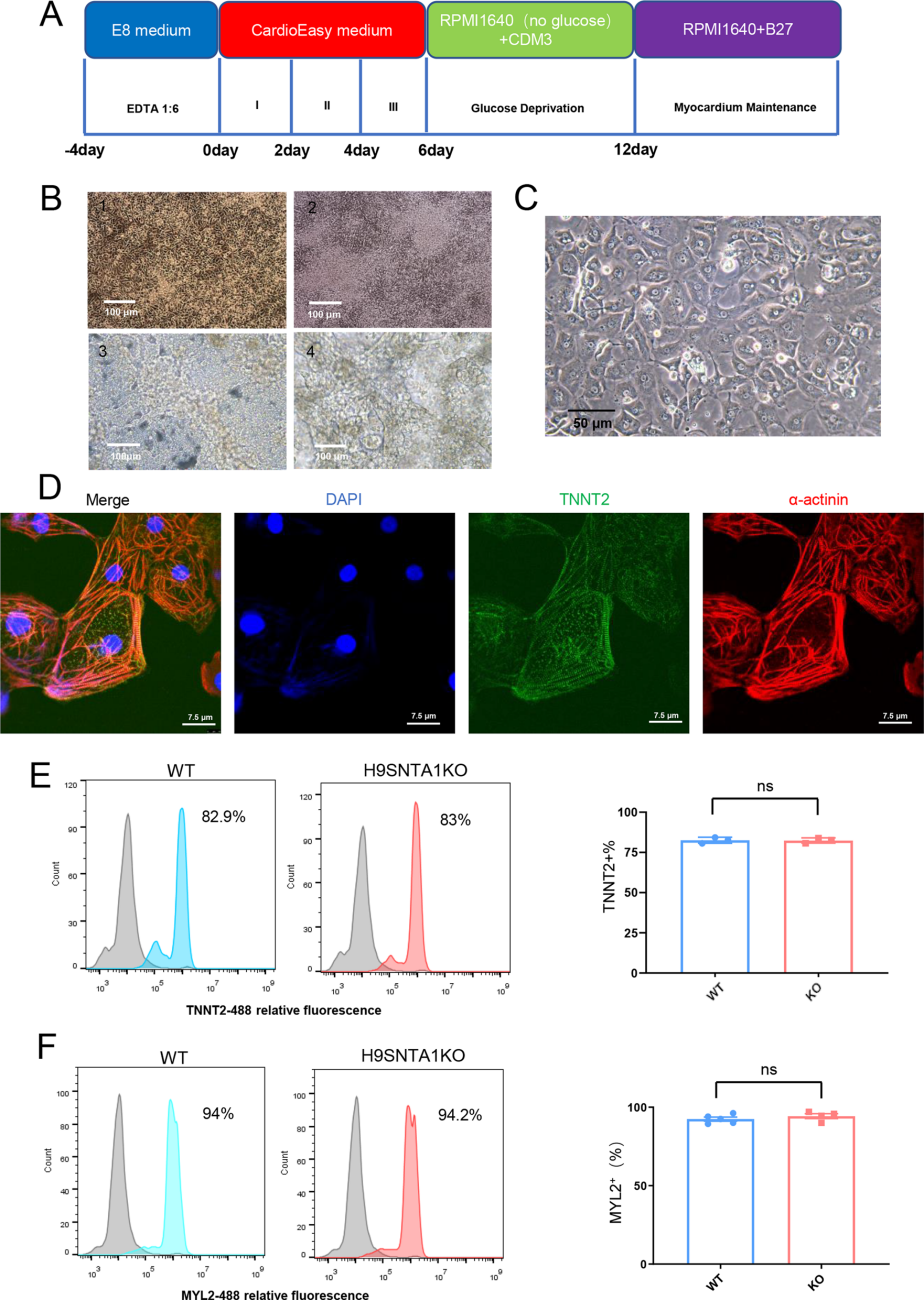
H9SNTA1KO differentiated into cardiomyocytes. **A** Schematic of hESCs induction into cardiomyocytes using small molecule inhibitors. **B** Image B1–B3 were hESCs induction into cardiomyocytes using working solutions. Scale bar: 100 μm. Image B4 showed the mass of beating cardiomyocytes on the 10th day of differentiation. Scale bar: 100 μm. **C** The image of KO exhibited purified by metabolic selection using a glucose-free medium supplemented with lactate. Scale bar: 50 μm. **D** Immunostaining of TNNT2 (green) and α-actinin (red) in KO. Scale bar: 7.5 μm. **E** The left graph: Flow cytometry was used to detect a specific cardiac marker, TNNT2. The result demonstrated that the differentiation rate of H9SNTA1KO was similar to the WT without purification. The right graph: Quantification of TNNT2 based on flow cytometry (n = 3). ns; not significant, unpaired two-sided Student’s *t* test. **F** The left graph: Flow cytometry was used to detect a specific ventricular muscle marker, MYL2. The results demonstrated that the yield of WT and KO was similarly purified using metabolic selection. The right graph: Quantification of MYL2 of the flow cytometry (n = 3). ns; not significant, unpaired two-sided Student’s *t* test

The process of H9 embryonic stem cells and H9SNTA1KO cells was induced into cardiomyocytes using the CardioEasy kit (Cellapy, China) (Fig. 2A). We recorded the process of H9SNTA1KO induction into cardiomyocytes. From Day 1 to Day 2, H9SNTA1KO was cultured in CardioEasy I medium (Fig. 2B1). From Day 3 to Day 4, H9SNTA1KO was cultured in CardioEasy II medium (Fig. 2B2). From Day 5 to Day 6, H9SNTA1KO was cultured in CardioEasy III medium (Fig. 2B3). On the 10th day of cardiac differentiation, we noticed the beating of KO-cardiomyocytes (Fig. 2B4). A glucose-free medium containing lactate was used to enhance KO-cardiomyocytes with metabolic selection. After cell density adjustment the KO-cardiomyocytes layer was formed (Fig. 2C). To identify the kind of differentiated cells, we stained the cells with TNNT2 and α-actinin cardiomyocytes markers. The results showed the beating cells, which differentiated from H9SNTA1KO, were positive for TNNT2 and α-actinin (Fig. 2D). Using a flow cytometry assay, we examined the expression of the TNNT2 in WT-cardiomyocytes and KO-cardiomyocytes before metabolic selection. The results indicated that H9SNTA1KO could differentiate into cardiomyocytes (Fig. 2E). MYL2, a specific marker of ventricular muscle, was detected in WT-cardiomyocytes and KO-cardiomyocytes after metabolic selection using flow cytometry assay. The results showed that the H9SNTA1KO had a normally differentiated cardiomyocyte subtype (Fig. 2F).

### Exhibiting activation of hypertrophy-associated genes in KO-cardiomyocytes

**Fig. 3:**
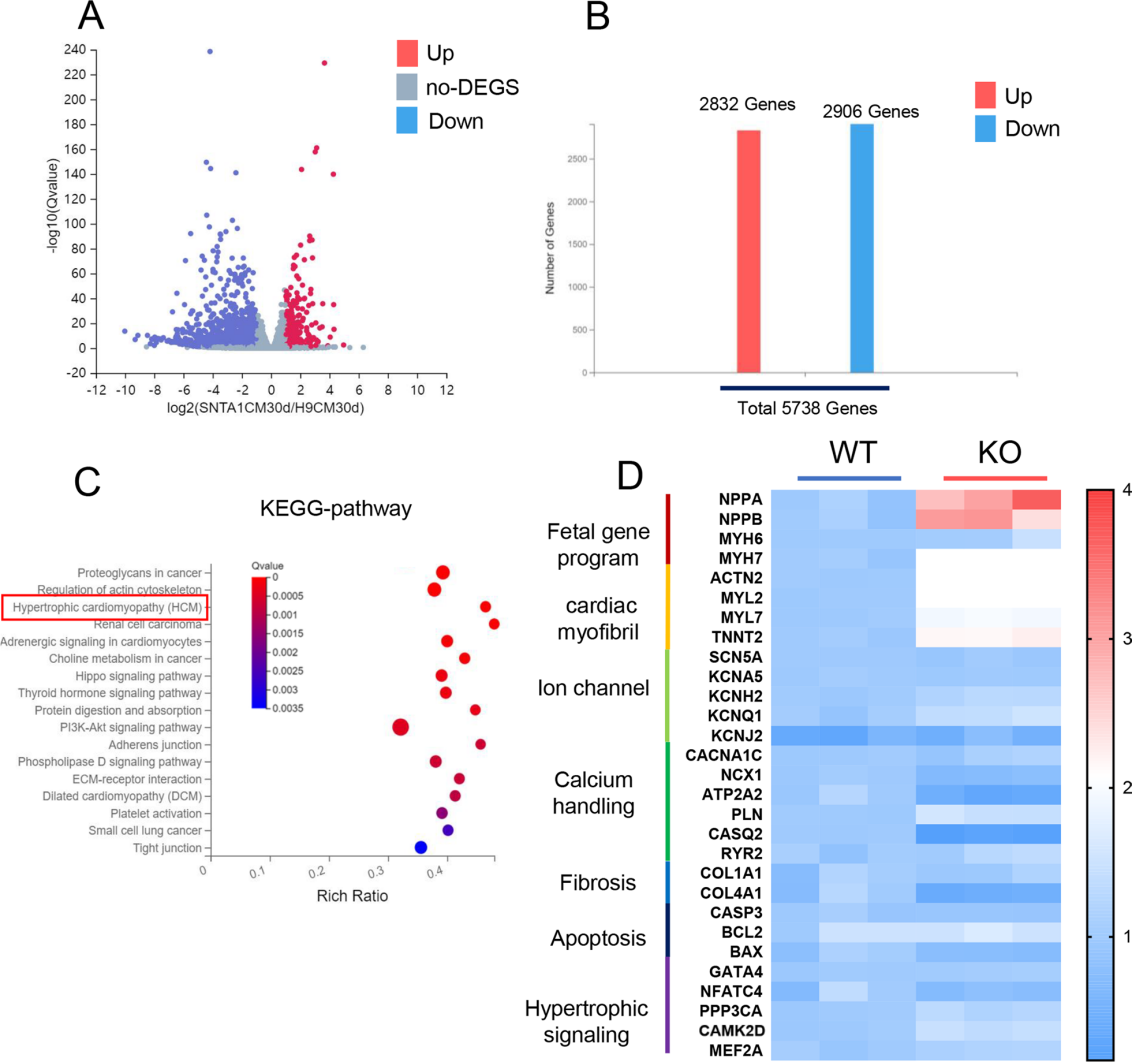
Exhibiting activation of hypertrophy-associated genes in KO-cardiomyocytes. **A** The volcano map showed the statistics of differentially expressed genes in WT and KO on the 30th day of cardiac differentiation using RNA-seq. **B** The chart showed the number of upregulated genes and the number of downregulated genes in RNA-seq analysis of the KO-cardiomyocytes. **C** A panel of KEGG enrichment analysis of differentially expressed transcripts in KO-cardiomyocytes. **D** Heatmap showed the discrepancies in the expression of genes involved in hypertrophy in WT-cardiomyocytes and KO-cardiomyocytes

To study the phenotype of KO-cardiomyocytes, the RNA of KO-cardiomyocytes was analyzed by the RNA-sequencing after being cultured on the 30th day. After analyzing the transcriptome data, the volcano map showed the up-and down-regulated genes (Fig. 3A). There were 5738 transcripts that were differently expressed between WT and KO-cardiomyocytes, with 2832 transcripts upregulated and 2906 transcripts downregulated (Fig. 3B). The hypertrophic cardiomyopathy pathway was found to be enriched in KO-cardiomyocytes after KEGG enrichment analysis of differentially expressed transcripts (Fig. 3C). To further explore the pathological process of cardiac hypertrophy in KO-cardiomyocytes, a panel of genes involved in hypertrophy was measured by qRT-PCR. The KO-cardiomyocytes presented remarkably varied mRNA expression of fetal gene program, cardiac myofibril, and calcium handling, which has been identified to be closely related to cardiac hypertrophy. (Fig. 3D) [30]. We focused on the expression of fetal gene program, cardiac myofibril, and calcium handling,

### Exhibiting hypertrophic phenotype in KO-cardiomyocytes

**Fig. 4:**
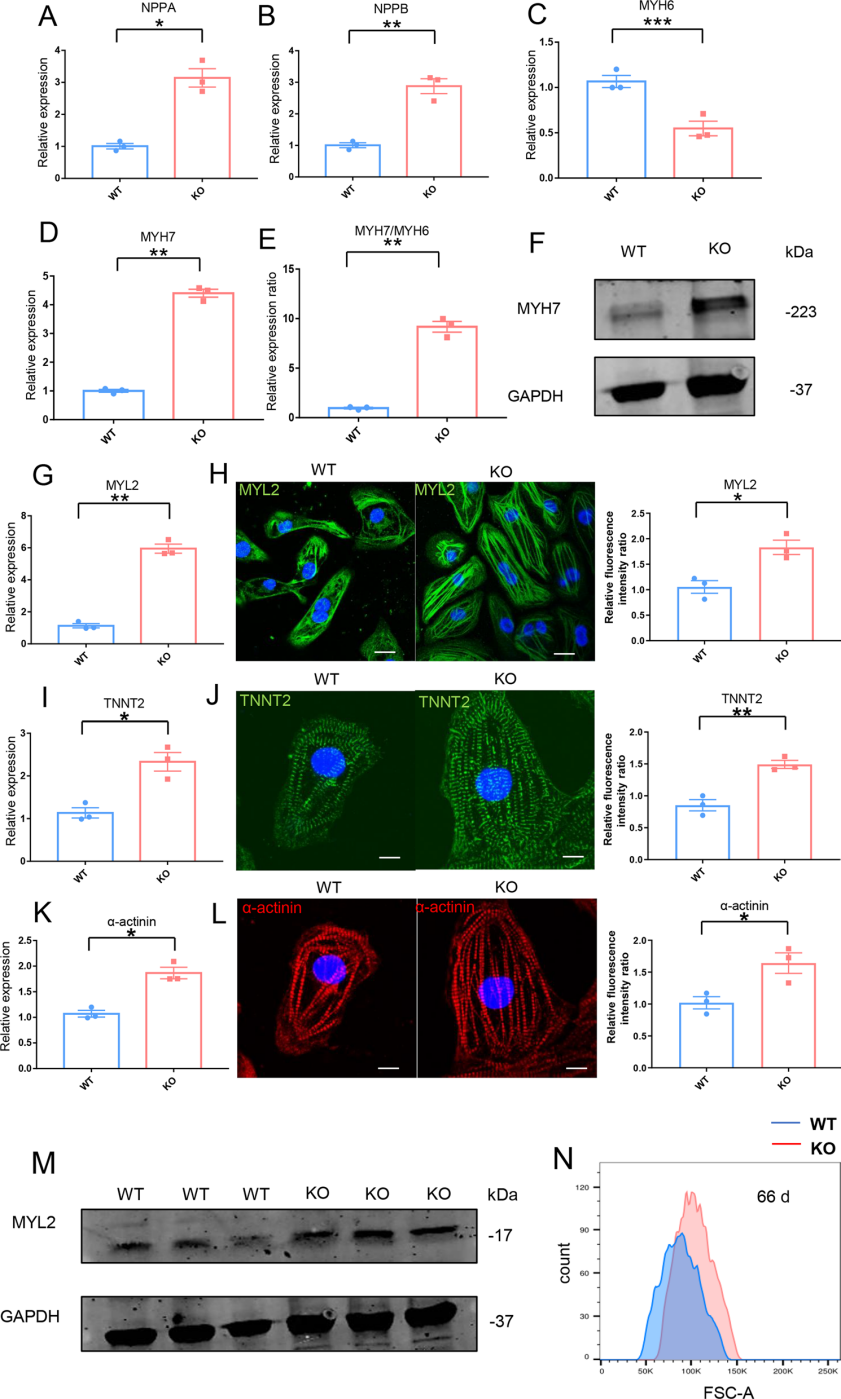
Exhibiting hypertrophic phenotype in KO-cardiomyocytes. **A** Comparison of NPPA at transcriptional level between WT-cardiomyocytes and KO-cardiomyocytes by qRT-PCR. n = 3 independent experiments. *P < 0.05. **B** Comparison of NPPB at transcriptional level between WT-cardiomyocytes and KO-cardiomyocytes by qRT-PCR. n = 3 independent experiments. **P < 0.01. **C** Comparison of MYH6 at transcriptional level between WT-cardiomyocytes and KO-cardiomyocytes by qRT-PCR. n = 3 independent experiments. ***P < 0.001. **D** Comparison of MYH7 at transcriptional level between WT-cardiomyocytes and KO-cardiomyocytes by qRT-PCR. n = 3 independent experiments. **P < 0.01. **E** Comparison of the ratio of MYH7 to MYH6 at transcriptional level between WT-cardiomyocytes and KO-cardiomyocytes. n = 3 independent experiments. **P < 0.01. **F** Immunoblot analysis of MYH7 in WT-cardiomyocytes and KO-cardiomyocytes. **G** Comparison of MYL2 at transcriptional level between WT-cardiomyocytes and KO-cardiomyocytes by qRT-PCR. n = 3 independent experiments. **P < 0.01. **H** Immunostaining of MYL2 (green) in WT-cardiomyocytes and KO-cardiomyocytes and semi-quantification analysis measured on the 45th day of cardiac differentiation. Scale bar: 10 μm. n = 3 independent experiments. *P < 0.05. **I** Comparison of TNNT2 at transcriptional level between WT-cardiomyocytes and KO-cardiomyocytes by qRT-PCR. n = 3 independent experiments. *P < 0.05. **J** Immunostaining of TNNT2 (green) in WT-cardiomyocytes and KO-cardiomyocytes and semi-quantification analysis measured on the 45th day of cardiac differentiation. Scale bar: 5 μm. n = 3 independent experiments. **P < 0.01. **K** Comparison of α-actinin at transcriptional level between WT-cardiomyocytes and KO-cardiomyocytes by qRT-PCR. n = 3 independent experiments. *P < 0.05. **L** Immunostaining of α-actinin (red) in WT-cardiomyocytes and KO-cardiomyocytes and semi-quantification analysis measured on the 45th day of cardiac differentiation. Scale bar: 5 μm. n = 3 independent experiments. *P < 0.05. **M** Immunoblot analysis of MYL2 in WT-cardiomyocytes and KO-cardiomyocytes. **N** The diameter size of WT-cardiomyocytes and KO-cardiomyocytes were evaluated using flow cytometry on the 66th day of cardiac differentiation

In cardiomyopathy, genes that are usually expressed during embryogenesis, such as natriuretic factors and β-MHC, are induced [31, 32]. Natriuretic factors include ANF and BNP. Hypertrophic cardiomyocytes frequently have higher levels of ANF and BNP expression [33].The expression of natriuretic factors was the focus of our research. qRT-PCR was used to detect the transcriptional levels of *ANF(NPPA)* and *BNP(NPPB)* in WT and KO-cardiomyocytes. The results showed the transcriptional level of *NPPA* and *NPPB* were increased in KO-cardiomyocytes (Fig. 4A, B). The expression of increased β-MHC influenced the myosin composition. In hypertrophic cardiomyopathy, myosin isoform shift is common [34]. qRT-PCR was used to detect the transcriptional levels of α-MHC (*MYH6*) and β-MHC (*MYH7*) in WT and KO-cardiomyocytes. The results indicated that the transcriptional level of *MYH6* was decreased and the *MYH7* was increased in KO-cardiomyocytes (Fig. 4C, D). The transcriptional ratio of MYH7 to MYH6 was increased (Fig. 4E). Then, using western blotting, the protein level of MYH7 in KO-cardiomyocytes was determined, and the results showed that the protein level of MYH7 increased (Fig. 4F). In KO-cardiomyocytes, myosin isoform change occurs. We also focused on the MYL2 and TNNT2, which are calcium-interacting components in cardiac myofibril. qRT-PCR was used to detect the transcriptional levels of *MYL2* and *TNNT2* in WT and KO-cardiomyocytes. The results showed that *MYL2* and *TNNT2* transcriptional levels were increased in KO-cardiomyocytes (Fig. 4G, I), and immunofluorescence staining with semi-quantitative analysis indicated that MYL2 and TNNT2 protein levels were increased in KO-cardiomyocytes (Fig. 4H, J). The *ACTN2*(α-actinin), which helps anchor the myofibrillar actin filaments was detected using qRT-PCR. The results showed that the transcription level of α-actinin was increased in KO-cardiomyocytes (Fig. 4K), and the protein of α-actinin was detected by immunofluorescence staining with semi-quantitative analysis. The results showed that the protein level of α-actinin was increased (Fig. 4L). We also used western blotting to detect MYL2 as a component of the myosin regulatory light chain, and the results showed that the protein level of MYL2 was increased (Fig. 4M). To summarize, there are increased cardiac myofibrillar components in KO-cardiomyocytes. We also used flow cytometry forward scatter (FSC) to measure the size of KO-cardiomyocytes and discovered that on the 66th day in vitro, the size of KO-cardiomyocytes increased (Fig. 4N). Overall, KO-cardiomyocytes showed a hypertrophic phenotype.

### The KO-cardiomyocytes exhibited calcium transient abnormality

**Fig. 5:**
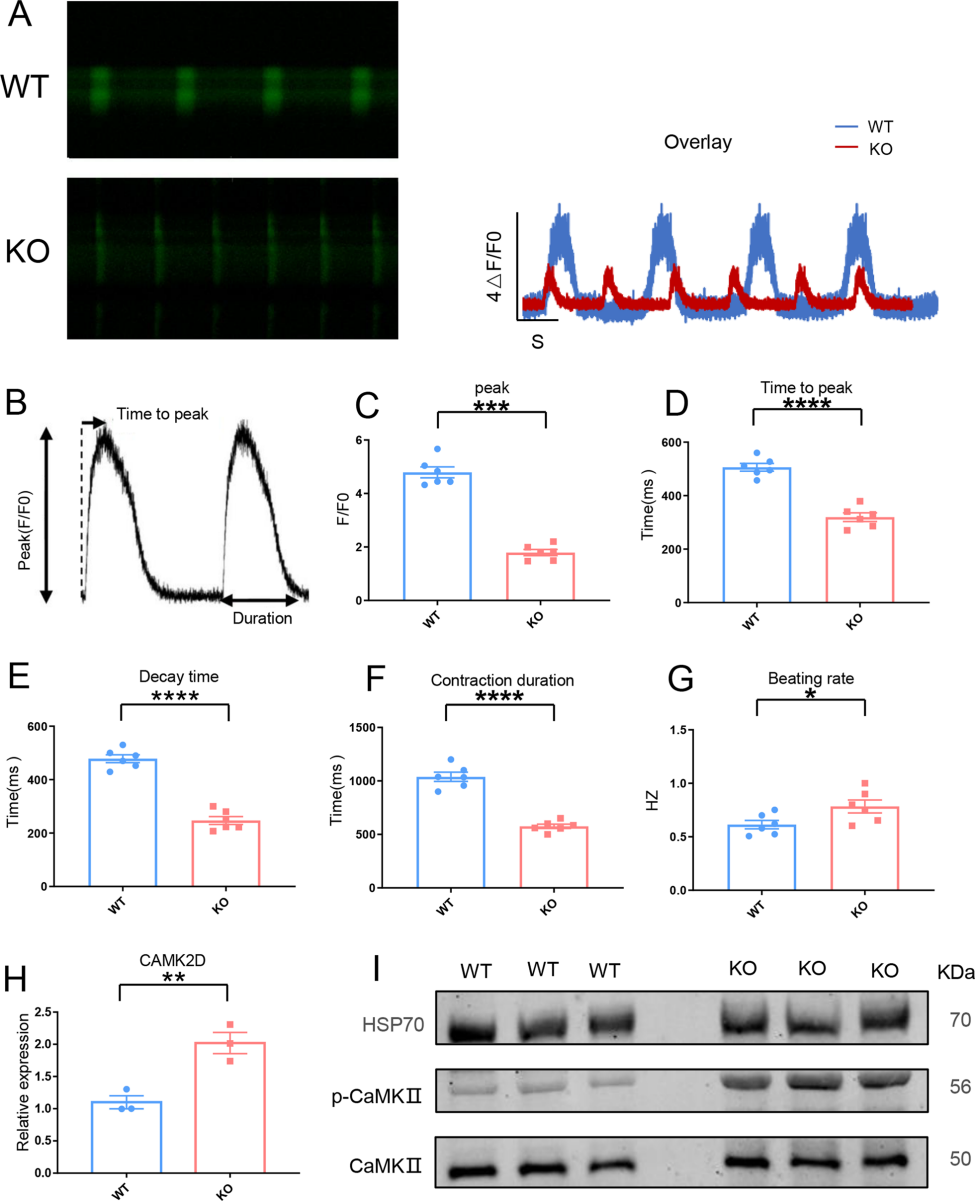
The KO-cardiomyocytes exhibited calcium transient abnormality. **A** The left panel: The representative line-scan image of WT-cardiomyocytes and KO-cardiomyocytes stained with Fluo-4. The right panel: Calcium transient profile derived from the left panel. **B** Space-averaged calcium transients showed parameters measured for analysis of calcium handling. **C**–**G** Quantification of peak, time to peak, decay time, contraction duration, and beating rate in WT-cardiomyocytes and KO-cardiomyocytes, n = 6. *P < 0.05; **P < 0.01; ***P < 0.001; ****P < 0.0001; unpaired two-sided Student’s *t* test. **H** Comparison of CAMK2D at transcriptional level between WT-cardiomyocytes and KO-cardiomyocytes by qRT-PCR. n = 3 independent experiments. **P < 0.01. **I** Immunoblot analysis of CaMKII in WT-cardiomyocytes and KO-cardiomyocytes

The KO-cardiomyocytes exhibited hypertrophic phenotype. The hypertrophic phenotype was usually associated with the abnormality of calcium handling [35–38]. For the reason stated above**,** we carried out a calcium transient test in WT-cardiomyocytes and KO-cardiomyocytes (Fig. 5A). The characteristics measured for calcium handling analysis are displayed as space-averaged calcium transients (Fig. 5B). The results showed that the peak value of calcium release was significantly decreased in KO-cardiomyocytes (Fig. 5C). In KO-cardiomyocytes, the time to peak and the time to decay of the calcium transient were both shorter (Fig. 5D, E, respectively). Furthermore, KO-cardiomyocytes had a shorter contraction duration (Fig. 5F). The KO-cardiomyocytes beating rate was increased as well. (Fig. 5G). All of these results confirmed that KO-cardiomyocytes intracellular calcium handling was abnormal. In cardiac myocytes, calcium/calmodulin-dependent kinase II (CaMKII) is a multifunctional serine/threonine kinase that is regulated by intracellular calcium [39]. Excessive CaMKII activation plays a pivotal role in the pathogenesis of severe heart conditions, including myocardial infarction, cardiomyopathy, and heart failure [40–42]. qRT-PCR was used to detect the transcriptional level of *CAMK2D* in WT-cardiomyocytes and KO-cardiomyocytes. The result showed the transcriptional level of *CAMK2D* was increased in KO-cardiomyocytes (Fig. 5H). Then, using western blotting, we detected the protein level of phosphorylated CaMKII (p-CaMKII) in WT-cardiomyocytes and KO-cardiomyocytes. The results showed that the protein level of p-CaMKII was elevated in KO-cardiomyocytes, indicating that KO-cardiomyocytes had excessive CaMKII activation (Fig. 5I). Overall, KO-cardiomyocytes showed the calcium transient abnormality.

### Abnormal caffeine-evoked Ca^2+^ release in KO-cardiomyocytes

**Fig. 6:**
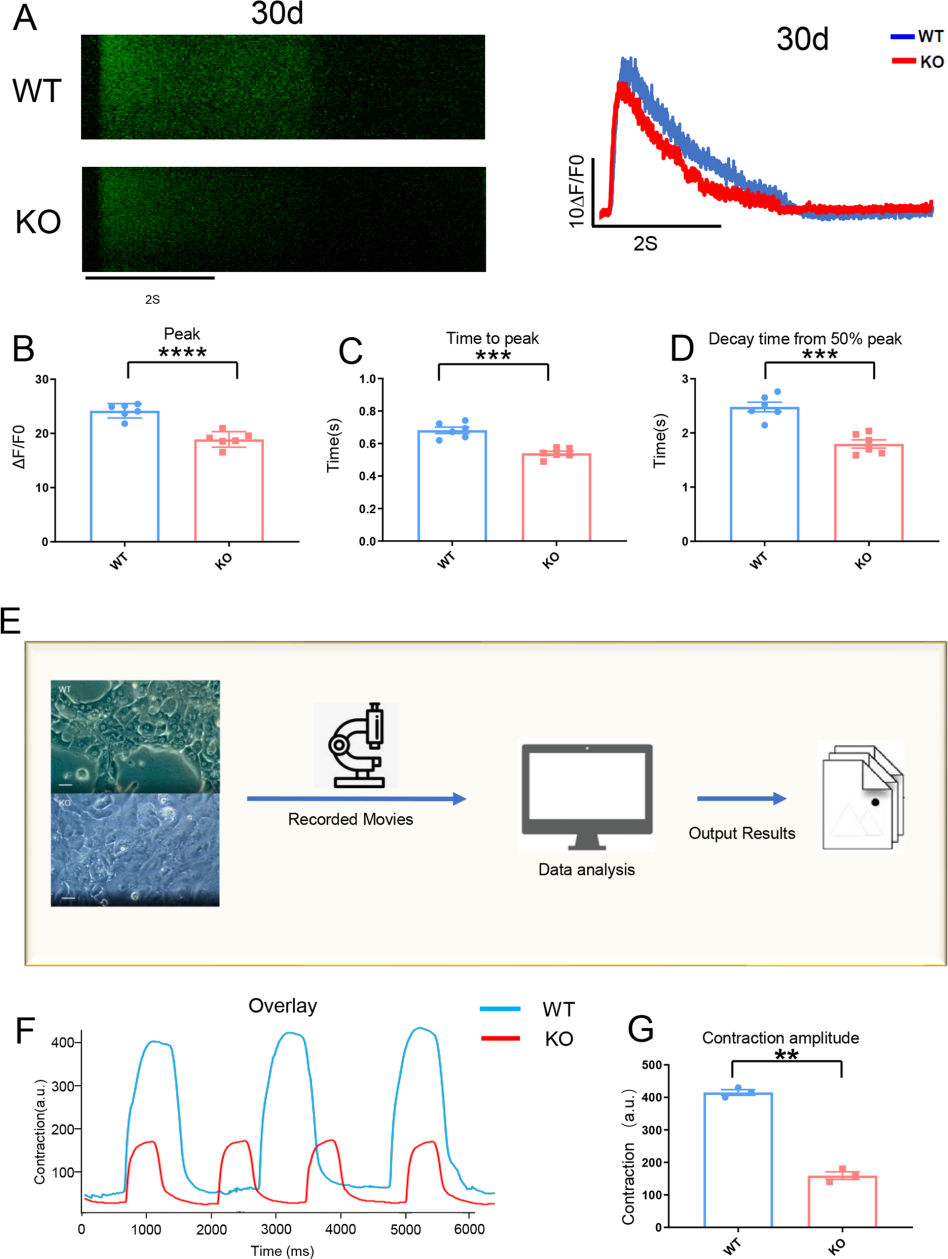
Abnormal caffeine-evoked Ca^2+^ release in KO-cardiomyocytes. **A** The left panel: The representative line-scan image of caffeine-evoked Ca^2+^ release in WT-cardiomyocytes and KO-cardiomyocytes stained with Fluo-4. The right panel: Ca^2+^ transients profile induced with 20 mM caffeine in Ca^2+^-free conditions derived from the left panel. **B**–**D** Quantification of peak, time to peak, and decay time in caffeine-evoked Ca^2+^ transients WT-cardiomyocytes and KO-cardiomyocytes, n = 6. ***P < 0.001; ****P < 0.0001; unpaired two-sided Student’s *t* test. **E** Schematic of the process of the contractility assess. **F** Contractility graph of WT-cardiomyocytes and KO-cardiomyocytes. **G** Quantification of contractility in WT-cardiomyocytes and KO-cardiomyocytes based on panel **F**. n = 3 independent experiments. **P < 0.01; unpaired two-sided Student’s *t* test

In KO-cardiomyocytes, intracellular calcium handling was disrupted, and the peak of calcium release decreased (Fig. 5). The SR, a calcium pool in cardiomyocytes, is directly related to the peak of intracellular calcium release. Therefore, we conducted caffeine-evoked Ca^2+^ release test on the KO-cardiomyocytes (Fig. 6A). The results indicated that the peak of caffeine-evoked calcium release was decreased in KO-cardiomyocytes (Fig. 6B) with the time of peak and decay time from 50% peak being shorter (Fig. 6C, D, respectively). The calcium ion level in SR was decreased in KO-cardiomyocytes compared with WT-cardiomyocytes. The release of calcium ion from SR partially influences the contractility of cardiomyocytes. The quantification of muscle contraction approach was used to assess the contractility of cardiac myocytes (Fig. 6E). The results showed that the amplitude of contraction was decreased in KO-cardiomyocytes compared with WT-cardiomyocytes (Fig. 6F, G, respectively).

### Impairment of calcium handling in KO-cardiomyocytes

**Fig. 7:**
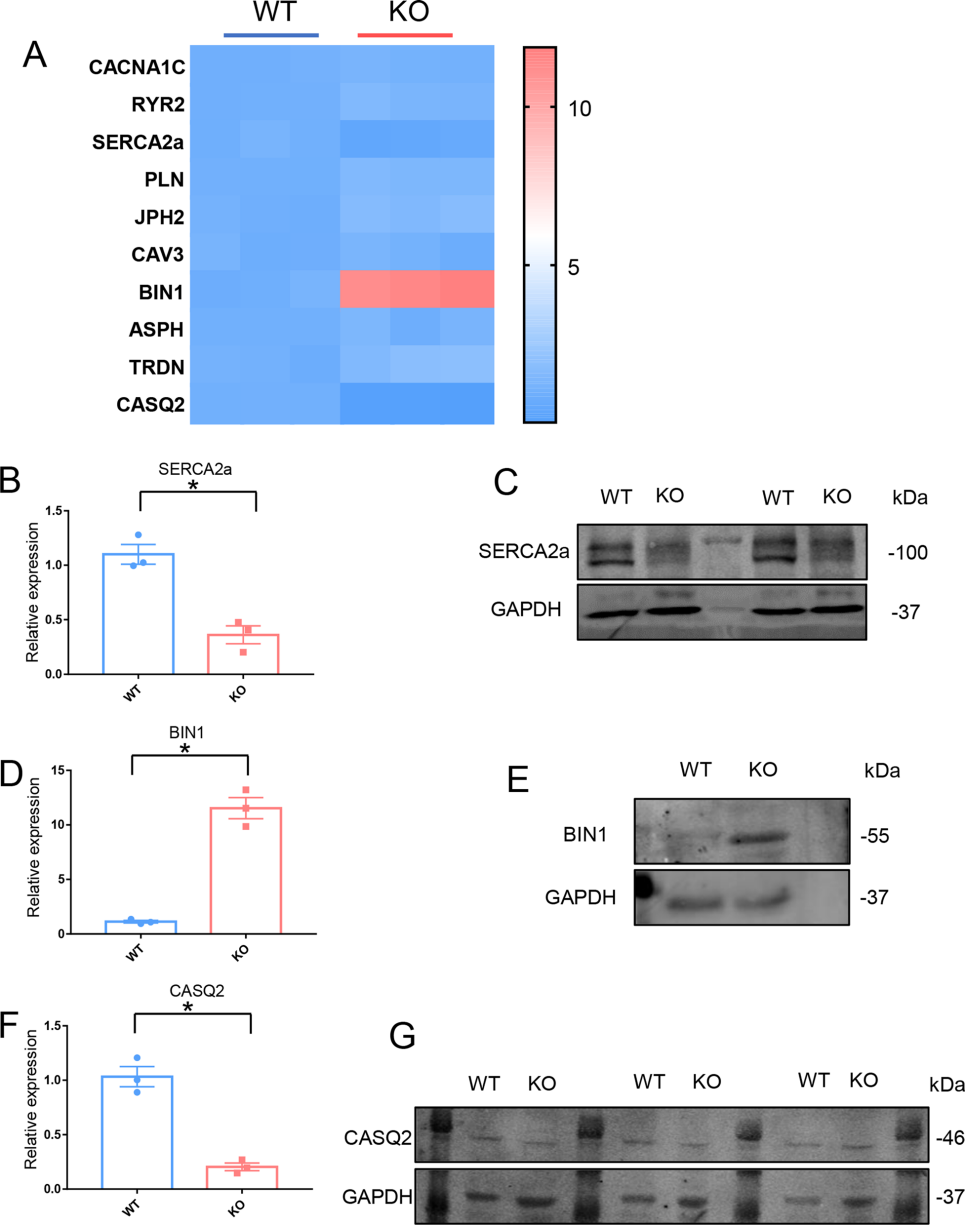
Impairment of calcium handling in KO-cardiomyocytes. **A** Heatmap showed the discrepancies in the expression of genes involved in calcium handling in WT-cardiomyocytes and KO-cardiomyocytes. **B** Comparison of SERCA2a at transcriptional level between WT-cardiomyocytes and KO-cardiomyocytes by qRT-PCR. n = 3 independent experiments. *P < 0.05. **C** Immunoblot analysis of SERCA2a in WT-cardiomyocytes and KO-cardiomyocytes. **D** Comparison of BIN1 at transcriptional level between WT-cardiomyocytes and KO-cardiomyocytes by qRT-PCR. n = 3 independent experiments. *P < 0.05. **E** Immunoblot analysis of BIN1 in WT-cardiomyocytes and KO-cardiomyocytes. **F** Comparison of CASQ2 at transcriptional level between WT-cardiomyocytes and KO-cardiomyocytes by qRT-PCR. n = 3 independent experiments. *P < 0.05. **G** Immunoblot analysis of CASQ2 in WT-cardiomyocytes and KO-cardiomyocytes

In KO-cardiomyocytes, we discovered abnormal calcium handling. In cardiomyocytes, the heatmap showed the transcriptional level of genes involved in calcium homeostasis (Fig. 7A). qRT-PCR was used to detect the transcriptional level of *SERCA2a,* which reuptake the cytoplasm calcium into SR in WT-cardiomyocytes and KO-cardiomyocytes. The result showed that the transcriptional level of *SERCA2a* was decreased in KO-cardiomyocytes (Fig. 7B). Western blotting was used to detect the protein level of SERCA2a. The results showed that the protein level of SERCA2a was decreased in KO-cardiomyocytes (Fig. 7C). BIN1 is a tubulogenesis membrane scaffolding protein that localizes the L-Type calcium channel to cardiac T-tubules and is a key regulator of EC coupling [22]. qRT-PCR was used to determine the transcriptional level of *BIN1* in WT-cardiomyocytes and KO-cardiomyocytes, the result showed that the transcriptional level of *BIN1* was elevated in KO-cardiomyocytes (Fig. 7D). Western blotting was used to detect the protein level of BIN1. The results showed that the protein level of BIN1 was elevated in KO-cardiomyocytes (Fig. 7E). The SR Ca^2+^-binding protein, CASQ2 plays an important role in the regulation of SR Ca^2+^ release by buffering Ca^2+^ in the SR [43]. qRT-PCR was used to detect the transcriptional level of *CASQ2* in WT-cardiomyocytes and KO-cardiomyocytes, the result showed that the transcriptional level of *CASQ2* was decreased in KO-cardiomyocytes (Fig. 7F). Western blotting was used to detect the protein level of CASQ2. The results showed the protein level of CASQ2 was decreased in KO-cardiomyocytes (Fig. 7G). Overall, in KO-cardiomyocytes there was a disorder of calcium handling protein expression.

### Early application of ranolazine improving the calcium handling of KO-cardiomyocytes

**Fig. 8:**
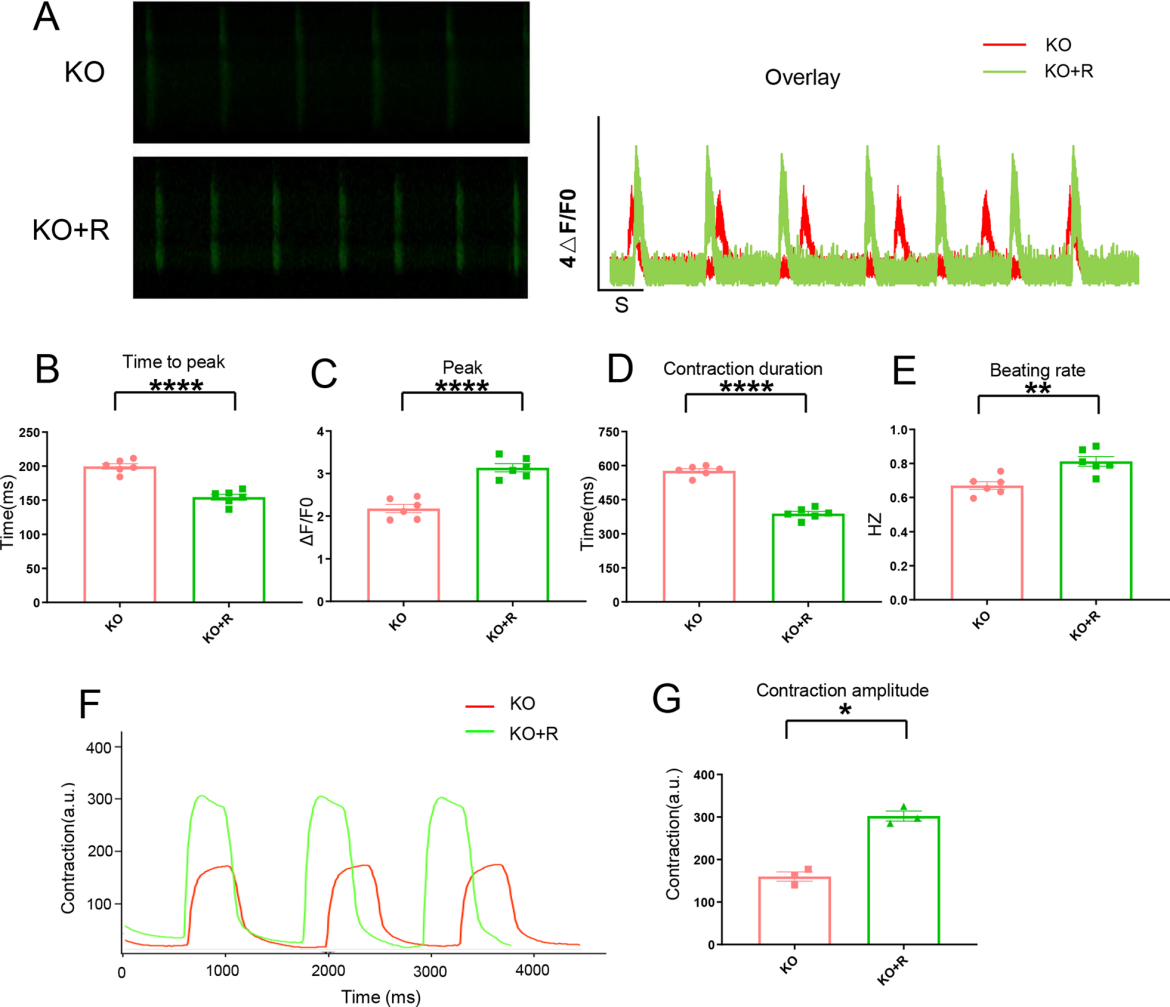
Early application of ranolazine improving the calcium handling of KO-cardiomyocytes. **A** The left panel: The representative line-scan image of the KO-cardiomyocytes and KO + R (KO-cardiomyocytes treated with 10 μM ranolazine) stained with Fluo-4 on the 20th day of cardiac differentiation. The right panel: Calcium transient profile derived from the left panel. **B**–**E**. Quantification of time to peak, peak, contraction duration, and beating rate in KO-cardiomyocytes and KO + R, n = 6. **P < 0.01; ****P < 0.0001; unpaired two-sided Student’s *t* test. **F** Contractility graph of KO-cardiomyocytes and KO + R (KO-cardiomyocytes treated with 10 μM ranolazine). **G** Quantification of the panel **F**. n = 3 independent experiments. *P < 0.05. Unpaired two-sided Student’s *t* test

Ranolazine usually relieve the chronic angina and is a well-tolerated medication that selectively inhibits the late sodium current [44], which works as an enhancer of the outward mode of the sodium-calcium exchanger (NCX) by blocking late sodium currents; hence, it indirectly promotes Ca^2+^ efflux while inhibiting fatty acid oxidation [45]. It has beneficial metabolic properties of hypertrophic cardiomyopathy [30]. Ranolazine (10 μM) was added on the 20th day cultured WT-cardiomyocytes and KO-cardiomyocytes for 24 h, respectively. Then, the calcium transient test was performed on the KO-cardiomyocytes (Fig. 8A). The result showed the time to peak of calcium transient test in KO-cardiomyocytes treated with ranolazine was shorter compared with no-treatment (Fig. 8B). The peak of the calcium transient test increased in KO-cardiomyocytes treated with ranolazine compared with no-treatment (Fig. 8C). The contraction duration became shorter in KO-cardiomyocytes treated with ranolazine compared with no-treatment (Fig. 8D). The beating rate was elevated in KO-cardiomyocytes treated with ranolazine compared with no-treatment (Fig. 8E). The results of the calcium transient test of WT-cardiomyocytes treated with ranolazine were shown in the Additional file 2: Fig. 2A-E. The results showed that early application of ranolazine elevated the peak of calcium release in KO-cardiomyocytes. We speculated that increasing calcium transient would ameliorate the contraction force. Hence, we detected the contraction force of KO-cardiomyocytes treated with ranolazine. The result showed the contractility force was increased in KO-cardiomyocytes treated with ranolazine (Fig. 8F, G, respectively). All the results showed that early treatment of ranolazine improved calcium release from SR in KO-cardiomyocytes.

## Discussion

**Fig. 9:**
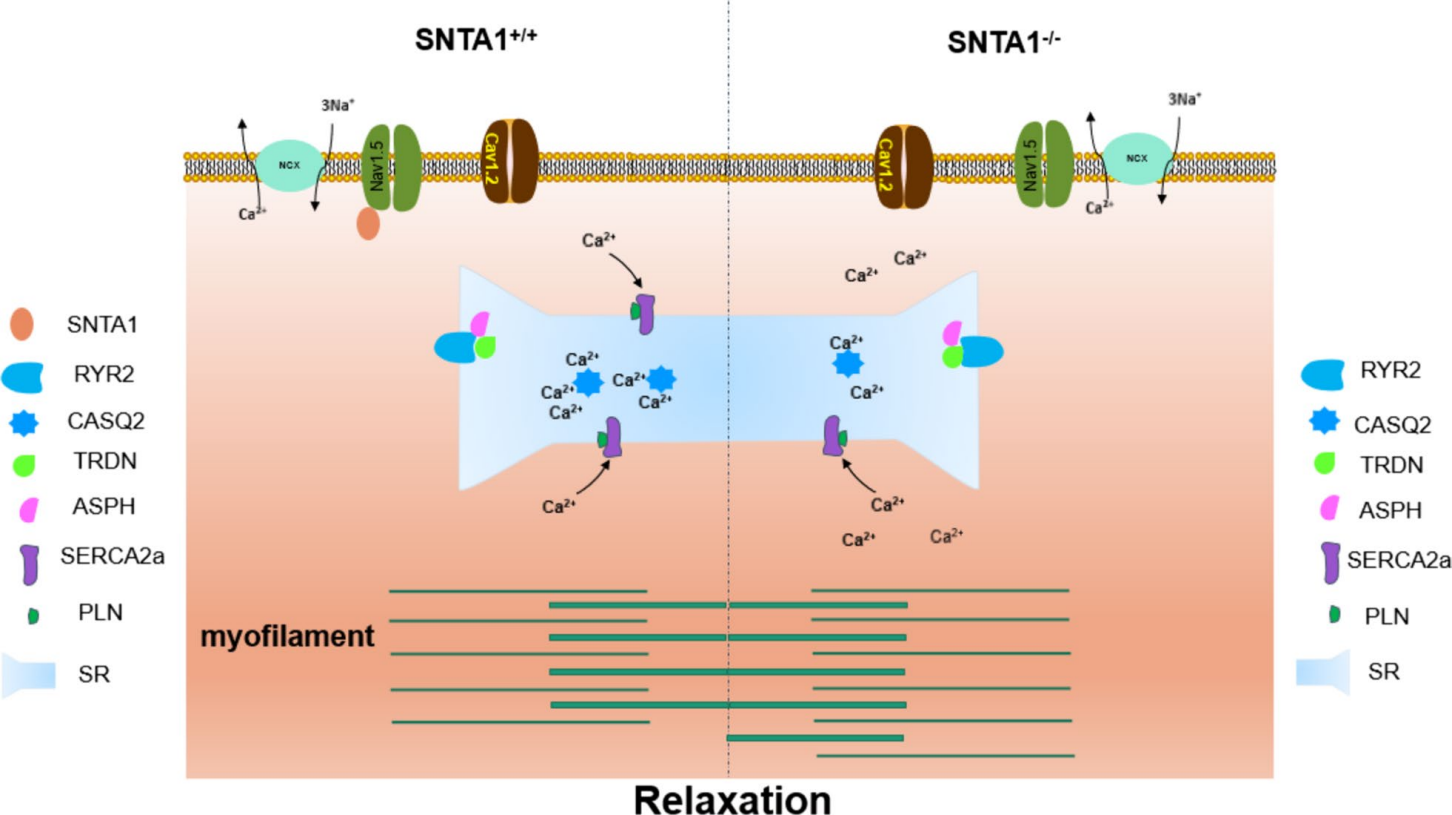
Schematic illustrating the disease model of KO-cardiomyocytes. Abnormal calcium handling in KO-cardiomyocytes

In the research, we reported a SNTA1-deficient cardiomyocyte model derived from SNTA1KO hESCs in vitro for the first time. Based on this cell model, dysfunction caused by SNTA1-deficiency could be well studied in the human cardiac background. After verification by multiple methods, we found that SNTA1-deficient cardiomyocytes exhibited hypertrophic phenotypes and disorder calcium handling. The abnormal regulation of intracellular calcium was the central mechanism of phenotypes (Fig. 9).

In human heart, SNTA1 is highly expressed. As a scaffold protein, SNTA1 binds to the C-terminal of Nav1.5 and played a vital regulatory role in Nav1.5 [2]. The PH1 domain and PDZ domain of SNTA1 have been reported to bind calmodulin [8] in a Ca^2+^-independent manner and a Ca^2+^-dependent manner [9]. Calmodulin transduces intracellular calcium signals by binding to calcium and then interacting with its downstream target proteins [10]. These interactions thus suggest a potential role of SNTA1 in calcium handling. Our data also demonstrated the vital role of SNTA1 in calcium handling.

Furthermore, transcriptomic discrepancies were investigated between WT-cardiomyocytes and KO-cardiomyocytes using RNA-seq after cardiomyocytes were purified using the metabolic method [46]. Analysis of RNA-seq data revealed that KO-cardiomyocytes had activation of genes associated with hypertrophy. qRT-PCR and western blotting analysis on genes associated with hypertrophy were performed on cardiomyocytes. On the 45th day of cardiac differentiation, the *ANF(NPPA)* and *BNP(NPPB)* were increased at the transcriptional level, which reflects the impaired function of cardiomyocytes caused by SNTA1 deficiency to some extent. ANF and BNP may be anti-hypertrophic to prevent overgrowth of the organ in response to stress, according to some data [47–49]. The *MYH7* and *MYH7*/*MYH6* were increased at the transcriptional level. Using western blotting, the protein level of MYH7 was increased in KO-cardiomyocytes. Our data showed there was a myosin isoform transformation in a cell model. This response may be adaptive, as MYH7 exhibits lower ATPase activity resulting in improved ATP use in KO-cardiomyocytes [30, 34]. Our data also showed the expression of MYL2, TNNT2, and α-actinin increased and increased cell size. Overall, all the phenotypes were associated with cardiac hypertrophy.

In cardiac myocytes, the phenotype of hypertrophy is usually associated with abnormal calcium handling [35, 36]. A calcium transient test was performed on KO-cardiomyocytes, and the results showed that the peak value of KO-cardiomyocytes was lower than the WT-cardiomyocytes (Fig. 5). The peak value of calcium transient is related to the level of calcium in the SR. The calcium loading in SR was measured using a caffeine-evoked calcium release test to elucidate the effect of SR function [50]. These results showed that the SR of calcium loading decreased in KO-cardiomyocytes. (Fig. 6). In cardiac disease development, intracellular calcium in the cardiomyocytes initially binds to calmodulin and then activates calcium-dependent cascades in the form of a calcium-calmodulin complex. Calcium-calmodulin-CaMKII pathways are major signal mediators of cardiac hypertrophy and remodeling [38, 41]. Our data showed p-CaMKII was increased, which represented the calcium-calmodulin-CaMKII pathway activation in KO-cardiomyocytes. This confirmed there were an abnormal calcium handling and cardiac hypertrophy in KO-cardiomyocytes.

The calcium release is related to the calcium loading in the SR. CASQ2, ASPH, and TRDN form complexes, which are involved in the SR calcium loading [51–53]. CASQ2 is a calcium-binding protein, which exists in the SR of cardiac muscle. It has low-affinity and high-capacity for binding calcium. CASQ2 is considered to be an essential protein for the storage and release of calcium in the SR [54]. The *Casq2-*knockdown mice only showed obvious SR calcium leak [55] and displayed early mortality [56]. The decrease of *CASQ2* expression in KO-cardiomyocytes led to the decrease in calcium loading in the SR during diastole. We hypothesized that decreased *CASQ2* expression may be disturbed by the disorder of calcium handling. Follow-up research will confirm the particular mechanism. Under the cellular membrane, SNTA1 is a scaffold protein [57]. Under the cellular membrane, BIN1 was another scaffold protein. We hypothesized the high expression of BIN1 was a kind of cellular compensatory protection, caused by SNTA1 deleted in KO-cardiomyocytes. In addition, the protein level of SERCA2a was decreased in KO-cardiomyocytes, which showed that the SR weakly reuptakes free calcium from the cytoplasm. In KO-cardiomyocytes, there was a decrease in the expression of CASQ2, an increase in the expression of BIN1, and a decrease in the expression of SERCA2a. In our cell model, there was an abnormal calcium homeostasis.

The metabolic support therapy could help to relieve the symptoms of cardiomyocyte diseases [58–60]. The metabolic support efforts alleviate the abnormal energy supply of hypertrophic, promote the survival rate, and improve myocardial cell functions [61]. Ranolazine inhibited the action of late sodium current, improved diastolic calcium overload, and the relaxation of ventricular myocytes [62, 63]. It also enhanced the NCX extroversion mode, indirectly promoted intracellular calcium excretion, and reduced high free intracellular calcium in diastole [30, 45]. On the 20th day of cardiac differentiation, we utilized 10 μM ranolazine to improve the phenotype of KO-cardiomyocytes. The results showed that the peak of calcium transient amplitude and the contraction force increased in KO-cardiomyocytes. Overall, the early application of ranolazine improved the phenotype of KO-cardiomyocytes.

## Conclusion

In the study, a human *SNTA1*-knockout cell model was established using the CRISPR-Cas9 system. The cell model can be used to study the dysfunction caused by the *SNTA1* deficiency in vitro. It provides a disease model for intracellular calcium homeostasis of cardiomyocytes study. It confirms that abnormal intracellular calcium handling was the core disease mechanism of *SNTA1*-deficient cardiomyocytes. This suggests that the strategy of maintenance of intracellular calcium homeostasis is a key target in the treatment of *SNTA1*-deficient myocardial diseases.

## Limitation

The cardiomyocytes we obtained had no T-tubules structure and that was unlike the mature cardiomyocytes. Our research was only performed in the two-dimensional (2D) cell culture in vitro.

## Supplementary Information

**Additional file 1.** The information of H9 embryonic stem cell line.

**Additional file 2.** Materials and Supplementary data.

## Data Availability

The RNA-seq data associated with this manuscript can be inquired from https://datadryad.org/stash/share/GHfhYHWUUS60ZellPwMfqLUNcziFeDla9zn2Yp9ie88.

## Abbreviations

SNTA1: α-1-Syntrophin
hESCs: Human embryonic stem cells
H9SNTA1KO: *SNTA1*-deficient H9 embryonic stem cells
WT: H9 embryonic stem cells
WT-cardiomyocytes: Cardiomyocytes derived from H9 embryonic stem cells
KO: *SNTA1*-Deficient H9 embryonic stem cells or H9SNTA1KO
KO-cardiomyocytes: *SNTA1*-Deficient cardiomyocytes
PAM: Protospacer adjacent motif
CRISPR-Cas9: Clustered regularly interspaced short palindromic repeats/CRISPR-associated protein 9
TNNT2: Troponin T2, cardiac type
SSEA4: Stage-specific embryonic antigen-4
NANOG: Nanog homeobox
SOX2: SRY-box transcription factor 2
DPPA4: Developmental pluripotency associated 4
OCT-4: POU class 5 homeobox 1
MYL2: Myosin light chain 2
KEGG: Kyoto encyclopedia of genes and genomes
MYH6: Myosin heavy chain 6
MYH7: Myosin heavy chain 7
ANF(NPPA): Natriuretic peptide A
BNP(NPPB): Natriuretic peptide B
p-CaMKII: P-Ca^2+^/calmodulin-dependent protein kinase II
CaMKII: Ca^2+^/calmodulin-dependent protein kinase II
EC: Excitation–contraction coupling
SR: Sarcoplasmic reticulum
CACNA1C: Calcium voltage-gated channel subunit alpha1 C
SERCA2a(ATP2A2): ATPase sarcoplasmic/endoplasmic reticulum Ca^2+^ transporting 2
BIN1: Bridging integrator 1
CASQ2: CASQ2
RyR2: Ryanodine receptor 2
CICR: Calcium-induced-calcium release
JPH2: Junctophilin 2
CAV3: Caveolin 3
NCX: Sodium-calcium exchanger
ASPH: Aspartate beta-hydroxylase
TRDN: Triadin

## Code availability

The RNA-seq data associated with this manuscript can be inquired from biosys.bgi.com (Username: F20FTSNCWLJ4149_PEOxzmR).
