# Supplementary material for: SNTA1-deficient human cardiomyocytes demonstrate hypertrophic phenotype and calcium handling disorder
Source: Stem Cell Res Ther. 2022 Jun 30;13:288. doi: 10.1186/s13287-022-02955-4 (PMC9248201; doi:10.1186/s13287-022-02955-4)
Supplement: Supplementary file 1 — Additional file 1. The information of H9 embryonic stem cell line. [file 13287_2022_2955_MOESM1_ESM.pdf]

Product Information and Testing - Amended

Product Information

Product Name	WA09 Research Bank
Alias	H9
Lot Number	WIC-WA09-RB-001
Parent Material	WIC-WA09-MB-001
Depositor	WiCell
Banked by	WiCell
Thaw Recommendation	Thaw 1 vial into 3 wells of a 6 well plate.
Culture Platform	Feeder Independent
	Medium: mTeSR1
	Matrix: Matrigel
Protocol	WiCell Feeder Independent Protocol
Passage Number	p30<br><br>These cells were cultured for 29 passages prior to freeze, 7 of them in mTeSR1/Matrigel. WiCell adds +1 to the passage number at freeze so that the number on the vial best represents the overall passage number of the cells at thaw.
Date Vialed	08-May-2009
Vial Label	WiCell WA09<br>Research Cell Bank<br>WIC WA09 RB 001<br>08-May-09
Biosafety and Use Information	Appropriate biosafety precautions should be followed when working with these cells. The end user is responsible for ensuring that the cells are handled and stored in an appropriate manner. WiCell is not responsible for damages or injuries that may result from the use of these cells.<br>Cells distributed by WiCell are intended for research purposes only and are not intended for use in humans.

Testing Performed by WiCell

Test Description	Test Provider	Test Method	Test Specification	Result
Post-Thaw Viable Cell Recovery	WiCell	SOP-CH-305	≥ 15 Undifferentiated Colonies,<br>≤ 30% Differentiation	Pass
Identity by STR	UW Molecular Diagnostics Laboratory	PowerPlex 1.2 System by Promega	Positive identity	Pass
Sterility - Direct transfer method	Apptec	30744	No contamination detected	Pass
Mycoplasma	Bionique	M250	No contamination detected	Pass
Karyotype by G-banding	WiCell	SOP-CH-003	Normal karyotype	Pass
Flow Cytometry for ESC Marker Expression	UW Flow Cytometry Laboratory	SOP-CH-101<br>SOP-CH-102<br>SOP-CH-103<br>SOP-CH-105	Report - no specification	See report

©2009 WiCell Research Institute The material provided under this certificate has been subjected to the tests specified and the results and data described herein are accurate based on WiCell's reasonable knowledge and belief. Appropriate Biosafety Level practices and universal precautions should always be used with this material. For clarity, the foregoing is governed solely by WiCell's Terms and Conditions of Service, which can be found at http://www.wicell.org/privacyandterms.

Product Information and Testing - Amended

Amendment(s):

Reason for Amendment	Date
CoA upated to include copyright information.	See Signature
CoA updated for format changes, addition of product information, and removal of footnotes.	13-Mary-2013
CoA updated to include text regarding research purposes only, include passage information, removed text regarding technical services, and update product description.	19-January-2011
CoA updated to reflect correct product description.	07-September-2010
CoA updated for format changes, clarification of test specifications, test method, addition of test provider, culture platform, and electronic signature.	10-JUN-2010
Original CoA	06-AUG-2009

Date of Lot Release	Quality Assurance Approval
06-August-2009	1/21/2014<br>X AMC<br>AMC<br>Quality Assurance<br>Signed by: [redacted]

©2009 WiCell Research Institute The material provided under this certificate has been subjected to the tests specified and the results and data described herein are accurate based on WiCell's reasonable knowledge and belief. Appropriate Biosafety Level practices and universal precautions should always be used with this material. For clarity, the foregoing is governed solely by WiCell's Terms and Conditions of Service, which can be found at http://www.wicell.org/privacyandterms.

UWHealth
University of Wisconsin
Hospital and Clinics

Histocompatibility/Molecular Diagnostics Laboratory

Short Tandem Repeat Analysis*

Sample Report: 2693-STR UW HLA#: 61145 Sample Date: 06/18/09
Received Date: 06/18/09

Requestor: WiCell Research Institute
Test Date: 06/23/09 File Name: 090624 Report Date: 06/25/09

Sample Name: **(label on tube)** **2693-STR**

Description: DNA Extracted by WiCell
267.58 ug/mL; 260/280 = 1.85

Locus	Repeat #	STR Genotype
D16S539	5, 8-15	12,13
D7S820	6-14	9,11
D13S317	7-15	9,9
D5S818	7-15	11,12
CSF1PO	6-15	11,11
TPOX	6-13	10,11
Amelogenin	NA	X,X
TH01	5-11	9.3,9.3
vWA	11, 13-21	17,17

Comments: Based on the DNA 2693-STR dated 06/18/09 and received on 06/18/09 from WI Cell, this sample (UW HLA# 61145) matches exactly the STR profile of the human stem cell line H9 comprising 12 allelic polymorphisms across the 8 STR loci analyzed. No STR polymorphisms other than those corresponding to the human H9 stem cell line were detected and the concentration of DNA required to achieve an acceptable STR genotype (signal/noise) was equivalent to that required for the standard procedure (~1 ng/amplification reaction) from human genomic DNA. These results suggest that the 2693-STR DNA sample submitted corresponds to the H9 stem cell line and it was not contaminated with any other human stem cells or a significant amount of mouse feeder layer cells. Sensitivity limits for detection of STR polymorphisms unique to either this or other human stem cell lines is ~5%.

6/29/09
Manager Date
HLA/Molecular Diagnostics Laboratory

06/29/09
PhD, Director Date
HLA/Molecular Diagnostics Laboratory

* Testing to assess engraftment following bone marrow transplantation was accomplished by analysis of human genetic polymorphisms at STR loci. This methodology has not yet been approved by the FDA and is for investigational use only.

File: Final STR Report

This report is confidential. No part may be used for advertising or public announcement without written permission. Results apply only to the sample(s) tested.

WuXi AppTec

WiCell Research Institute

June 04, 2009
P.O. #:

STERILITY TEST REPORT

Sample Information: hES Cells, WIC-WA09-RB001 #4208

Date Received: May 19, 2009
Date in Test: May 20, 2009
Date Completed: June 03, 2009

Test Information: Test Codes: 30744, 30744A
Immersion, USP / 21 CFR 610.12
Procedure #: BS210WCR.201

TEST PARAMETERS	PRODUCT	
Approximate Volume Tested	0.5 mL	0.5 mL
Number Tested	2	2
Type of Media	SCD	FTM
Media Volume	400 mL	400 mL
Incubation Period	14 Days	14 Days
Incubation Temperature	20 °C to 25 °C	30 °C to 35 °C
RESULTS	2 NEGATIVE	2 NEGATIVE

QA Reviewer — Date: 06-05-09

Technical Reviewer — Date: 06-05-09

Testing conducted in accordance with current Good Manufacturing Practices.

Mycoplasma Testing Services

Safe Cells

BIONIQUE TESTING LABORATORIES, INC.

Document#:	DCF3013D
Edition#:	10
Effective Date:	07/15/2003
Title:	**M-250 FINAL REPORT SHEET**

M-250 FINAL REPORT

Direct Specimen Culture
Procedure 3008, 3011, 3013

TO: Wicell QA

BTL SAMPLE ID#: **57619** P.O.#: DATE REC'D: **06/03/2009**

TEST/CONTROL ARTICLE:

WIC-WA09.RB-001-B

LOT#: **#2693**

DIRECT CULTURE SET-UP (DAY 0) DATE: **06/03/2009**

INDICATOR CELL LINE (VERO) SEE DNA FLUOROCHROME RECORD SHEET

				DATE
THIOGLYCOLLATE BROTH	DAY 7	+	-	**06/10/2009**
	DAY 28	+	-	**07/01/2009**
BROTH-FORTIFIED COMMERCIAL				
**0.5** mL SAMPLE	DAY 7	+	-	**06/10/2009**
**6.0** mL BROTH	DAY 28	+	-	**07/01/2009**
BROTH-MODIFIED HAYFLICK				
**0.5** mL SAMPLE	DAY 7	+	-	**06/10/2009**
**6.0** mL BROTH	DAY 28	+	-	**07/01/2009**
BROTH-HEART INFUSION				
**0.5** mL SAMPLE	DAY 7	+	-	**06/10/2009**
**6.0** mL BROTH	DAY 28	+	-	**07/01/2009**

(See Reverse)

APPENDIX IV

Document#:	DCF3013D
Edition#:	10
Effective Date:	07/15/2003
Title:	M-250 FINAL REPORT SHEET

SAMPLE ID#: **57619**		AEROBIC	MICROAEROPHILIC	DATE
AGAR PLATES-FORTIFIED COMMERCIAL	DAY 7	+ ⊖	+ ⊖	06/10/2009
	DAY 14	+ ⊖	+ ⊖	06/17/2009
	DAY 21	+ ⊖	+ ⊖	06/24/2009
AGAR PLATES-MODIFIED HAYFLICK	DAY 7	+ ⊖	+ ⊖	06/10/2009
	DAY 14	+ ⊖	+ ⊖	06/17/2009
	DAY 21	+ ⊖	+ ⊖	06/24/2009
AGAR PLATES-HEART INFUSION	DAY 7	+ ⊖	+ ⊖	06/10/2009
	DAY 14	+ ⊖	+ ⊖	06/17/2009
	DAY 21	+ ⊖	+ ⊖	06/24/2009

BROTH SUBCULTURES (DAY 7) DATE: 06/10/2009

		AEROBIC	MICROAEROPHILIC	DATE
AGAR PLATES-FORTIFIED COMMERCIAL	DAY 7	+ ⊖	+ ⊖	06/17/2009
	DAY 14	+ ⊖	+ ⊖	06/24/2009
	DAY 21	+ ⊖	+ ⊖	07/01/2009
AGAR PLATES-MODIFIED HAYFLICK	DAY 7	+ ⊖	+ ⊖	06/17/2009
	DAY 14	+ ⊖	+ ⊖	06/24/2009
	DAY 21	+ ⊖	+ ⊖	07/01/2009
AGAR PLATES-HEART INFUSION	DAY 7	+ ⊖	+ ⊖	06/17/2009
	DAY 14	+ ⊖	+ ⊖	06/24/2009
	DAY 21	+ ⊖	+ ⊖	07/01/2009

RESULTS: No detectable mycoplasmal contamination

7-1-09
Date

[illegible]
Laboratory Director
Ph.D.

M-250 Procedural Summary: The objective of this test is to ascertain whether or not detectable mycoplasmas are present in an in vitro cell culture sample, be it a primary culture, hybridoma, master seed stock or cell line. This procedure combines an indirect DNA staining approach to detect non-cultivable mycoplasmas with a direct culture methodology utilizing three different mycoplasmal media formulations. The indirect approach involves the inoculation of the sample into a mycoplasma-free VERO (ATCC) indicator cell line and performing a DNA fluorochrome assay after 72-120 hours of incubation. The direct culture aspect of the test utilizes three different mycoplasmal media including both broth and agar formulations. The sample is inoculated into each of the 3 broth formulations and also onto duplicate plates (0.1 mL/plate) for each of the 3 agar formulations. Subculture from broth to fresh agar plates is carried out after 7 days incubation. Agar plates are incubated aerobically and microaerophillically in order to detect any colony forming units morphologically indicative of mycoplasmal contamination. Issuance of the final report with signature of the Laboratory Director signifies that the required controls were performed concurrently with the test sample(s) as detailed in the referenced SOPs and that all test conditions have been found to meet the required acceptance criteria for a valid test, including the appropriate results for the positive and negative controls.

APPENDIX I

Document #: DCF3008A
Edition #: 06
Effective date: 9/17/2003
Title: DNA FLUOROCHROME ASSAY RESULTS

DNA-FLUOROCHROME ASSAY RESULTS

Procedures 3008, 3009, 3011

Sample ID # **57619** **M-250** Date Rec'd: **06/03/2009** P.O. #

Indicator Cells Inoculated: Date/Initials: 6/4/09 / KG

Fixation: Date/Initials: 6/8/09 / KG

Staining: Date/Initials: 6/8/09 / KG

TEST/CONTROL ARTICLE:

WIC-WA09.RB-001-B

LOT# **#2693**

Wicell OA

DNA FLUOROCHROME ASSAY RESULTS:

X **NEGATIVE:** A reaction with staining limited to the nuclear region, which indicates no mycoplasmal contamination.

___ **POSITIVE:** A significant amount of extranuclear staining which strongly suggests mycoplasmal contamination.

___ **INCONCLUSIVE:**

___ A significant amount of extranuclear staining consistent with low - level mycoplasmal contamination or nuclear degeneration.

___ A significant amount of extranuclear staining consistent with bacterial, fungal or other microbial contaminant or viral CPE. Morphology not consistent for mycoplasmal contamination.

COMMENTS:

Date: 6/8/09 Results Read by: KG Date of Review: 6/8/09 Reviewed by: [illegible]

WiCell Cytogenetics Report: 001097-052009
WISC 2693

Report Date: May 29, 2009

**Case Details:**

Cell Line: *WIC-WA09-RB-001 (2693)*

Passage #: *31*

Date Completed: *5/29/2009*

Cell Line Gender: *Female*

Investigator:

Specimen: *hESC on Matrigel*

Date of Sample: *5/20/2009*

Tests,Reason for: *GMP Research Bank Testing*

Results: *46,XX*

Completed by , CLSp(CG), on 5/29/2009

Reviewed and interpreted by , PhD, FACMG, on 5/29/2009

Interpretation: *No clonal abnormalities were detected at the stated band level of resolution.*

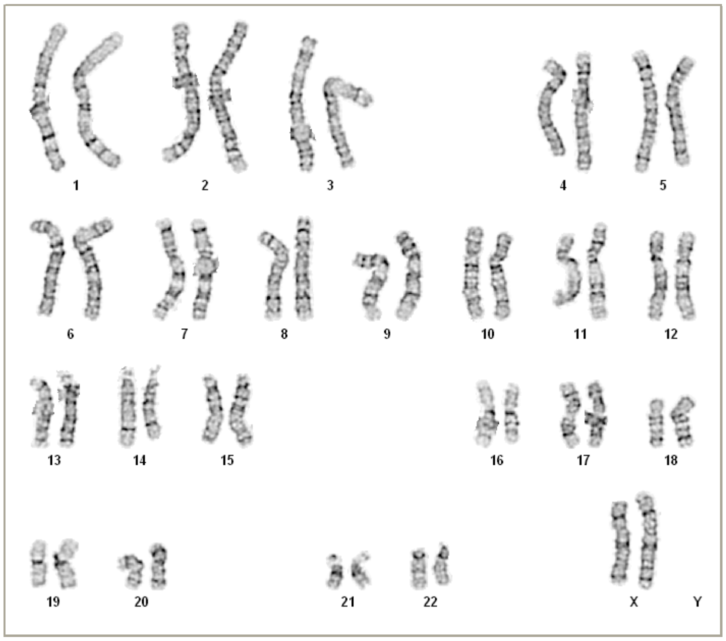

Cell: *S01-01*

Slide: *A*

Slide Type: *Karyotyping*

Cell Results: *Karyotype: 46,XX*

# of Cells Counted: 20

# of Cells Karyotyped: 4

# of Cells Analyzed: 8

Band Level: 450-550

Results Transmitted by Fax / Email / Post
Sent By:______________________________

Date:____________________________
Sent To:__________________________

Procedures performed:	Cell Line: WIC-WA09-RB-001	Date of: (mm/dd/yy)
SOP-CH-101 SOP-CH-102	Passage	acquisition: 06/10/09
SOP-CH-103 SOP-CH-105	Sample ID: 2693-FAC	file creation: 06/12/09
		file submission: 06/12/09

antigen2:	SSEA4 - antigen2 +	SSEA4 + antigen2 +	SSEA4 + antigen2 -	SSEA4 - antigen2 -	ALL SSEA4 +	ALL antigen2 +
SSEA3	2.63	96.30	0.60	0.46	96.90	98.93
TRA1-60	1.46	97.00	1.32	0.25	98.32	98.46
TRA1-81	0.20	98.50	1.24	0.05	99.74	98.70
Oct-4*	0.34	97.30	2.08	0.28	99.38	97.64
SSEA1	0.01	0.43	98.50	1.09	98.93	0.44

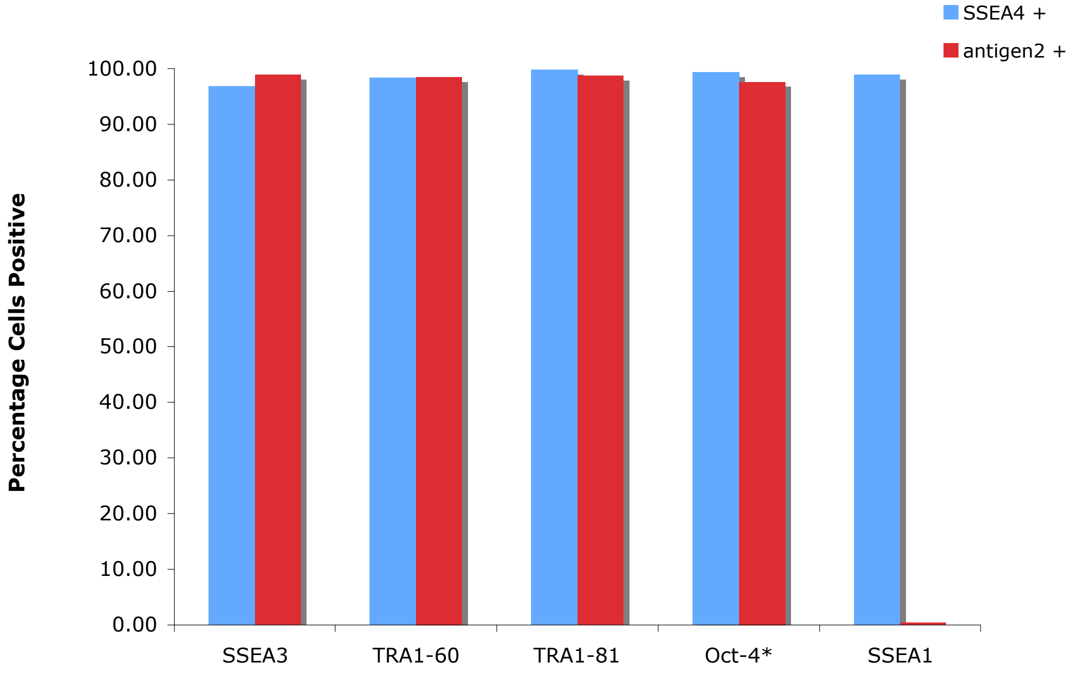

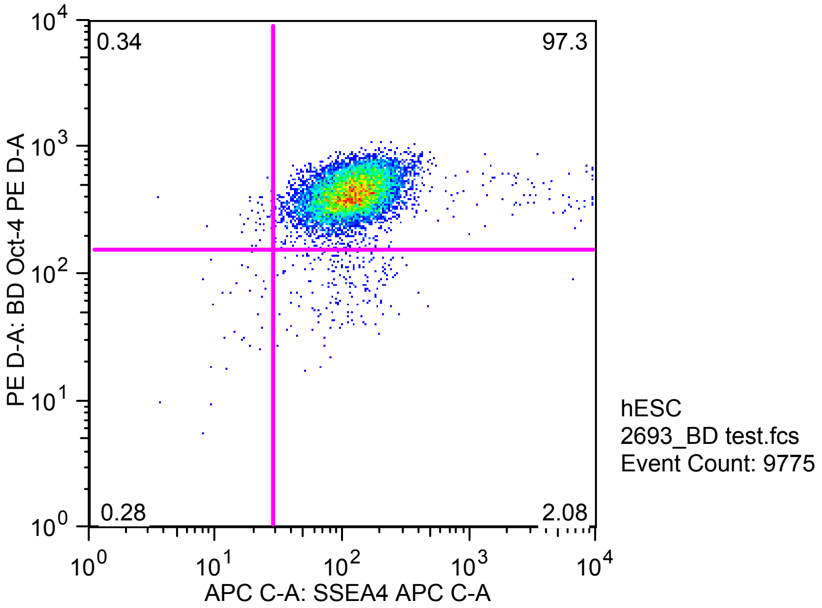

*PE-conjugated Oct-3/4 from BD Biosciences was used (cat #560186).
