# Supplementary material for: SNTA1-deficient human cardiomyocytes demonstrate hypertrophic phenotype and calcium handling disorder
Source: Stem Cell Res Ther. 2022 Jun 30;13:288. doi: 10.1186/s13287-022-02955-4 (PMC9248201; doi:10.1186/s13287-022-02955-4)
Supplement: Supplementary file 2 — Additional file 2. Materials and Supplementary data. [file 13287_2022_2955_MOESM2_ESM.pdf]

Table S1. Primer sequences used for q-PCR

Gene	Forward 5'-3'	Reverse 5'-3'
NPPA	ACAATGCCGTGTCCAACGCAGA	CTTCATTCGGCTCACTGAGCAC
NPPB	TCTGGCTGCTTTGGGAGGAAGA	CCTTGTGGAATCAGAAGCAGGTG
MYH6	TCTCCGACAACGCCTATCAGTAC	GTCACCTATGGCTGCAATGCT
MYH7	GGCAAGACAGTGACCGTGAAG	CGTAGCGATCCTTGAGGTTGTA
ACTN2	GAGGGCAAGATGGTGTCGGATA	CTTCTCAGCCAGGTGTTCCAAG
MYL2	TACGTTCGGGAAATGCTGAC	TTCTCCGTGGGTGATGATG
MYL7	CCGTCTTCCTCACGCTCTT	TGAACTCATCCTTGTTCACCAC
TNNT2	TTCACCAAAGATCTGCTCCTCGCT	TTATTACTGGTGTGGAGTGGGTGTGG
SCN5A	CTGACCTCACCATCACTATGTG	GCTGTGAAAATCCCTGTGAAG
KCNA5	GTTCCGCATCTTCAAGCTCTCC	CGAAGTAGACGGCACTGGAGAA
KCNH2	AATCGCCTTCTACCGGAAAG	CACCATGTCCTTCTCCATCAC
KCNQ1	TCTGTCTTTGCCATCTCCTTC	CCTCCATGCGGTCTGAATG
KCNJ2	AAGACGGTATGAAGTTGGCC	CGGGTGTGGACTTTACTCTTC
CACNA1C	CAGAGGCTACGATTTGAGGA	GCTTCACAAAGAGGTCGTGT
NCX1	TCGAGATTGTCTCTTCGGGC	CATATGCAAACACCGAGGCG
ATP2A2	GATCACACCGCTGAATCTG	AGTATTGCGGGTTGTTCCAG
PLN	AGCACGTCAAAAGCTACAGAATCT	CTGATGTGGCAAGCTGCAGATC
CASQ2	GCAGCAAACTGGAAGTCCAAGC	GATGTAAGGCTGGAAGTGTTCAG
BIN1	CGTCAACACGTTCCAGAGCATC	CTTGACCGTGAAGGTGTTGCTC
JPH2	CAGGAGTCCAACATTGCTCGCA	CTCTCCGAGTTCTCCAGGATCT
CAV3	GATTGACCTGGTGAACCGAGAC	CTTGGAGACAGTGAAGGTGGTG
ASPH	AGCCATCGAGACCTACCAAGAG	AGGGTAAGCAGGGAACCTCTCA
TRDN	GGAGGACAAAGAGAAAGCAGCTG	AGGTGGAATGGCTGGGCTTTGT
RYR2	AGAACTTACACACGCGACCTG	CATCTCTAACCGGACCATACTGC
COL1A1	GATTCCCTGGACCTAAAGGTGC	AGCCTCTCCATCTTTGCCAGCA
COL4A1	TGTTGACGGCTTACCTGGAGAC	GGTAGACCAACTCCAGGCTCTC
CASP3	GGTATTGAGACAGACAGTGG	CATGGGATCTGTTTCTTTGC

BCL2	ATCGCCCTGTGGATGACTGAGT	GCCAGGAGAAATCAAACAGAGGC
BAX	TCAGGATGCGTCCACCAAGAAG	TGTGTCCACGGCGGCAATCATC
GATA4	GCGGTGCTTCCAGCAACTCCA	GACATCGCACTGACTGAGAACG
NFATC4	GCACCGTATCACAGGCAAGATG	TCAGGATTCCCGCGCAGTCAAT
PPP3CA	GCCCTGATGAACCAACAGTTCC	GCAGGTGGTTCTTTGAATCGGTC
CAMK2D	ACACGGTGACTCCTGAAGCCAA	GTCTCCTGTCTGTGCATCATGG
MEF2A	CAAGGGCATGATGCCTCCACTA	GCTGAGTACACAAGTCCTTGCG
GAPDH	GGAGCGAGATCCCTCCAAAAT	GGCTGTTGTCATACTTCTCATGG

Table S2. Primary and Secondary Antibodies

Type	Antibody	Application	Dilution	Species	Manufacturer And Catalog Number
Primary	Anti-OCT4	Immunofluorescence	1:100	mouse monoclonal	Santa Cruz sc-5279
	Anti-SSEA4	Immunofluorescence	1:100	Mouse Monoclonal	Santa Cruz sc-21704
	Anti-cTnT	Immunofluorescence; Flow cytometry	1:100	Mouse Monoclonal	Abcam Ab8295
	Anti-MYL2	Immunofluorescence; Flow cytometry; Western blot	1:100; 1:100; 1:1000	Rabbit Polyclonal	Proteintech 10906-1-AP
	Anti-MYH7	Western blot	1:1000	Mouse Monoclonal	Abcam Ab 174640
	Anti-α-actinin	Immunofluorescence	1:100	Rabbit Polyclonal	Abcam Ab137346
	Anti-Phospho-CaMKII (Thr286)	Western blot	1:1000	Rabbit Monoclonal	Cell signaling #12716
	Anti-CaMKII	Western blot	1:1000	Rabbit Monoclonal	Abcam Ab52476
	Anti-GAPDH	Western blot	1:1000	Mouse Monoclonal	Santa Cruz sc-365062
	Anti-HSP70	Western blot	1:1000	Rabbit Polyclonal	Proteintech 10995-1-AP
	Anti-SERCA2a	Western blot	1:1000	Rabbit Monoclonal	Cell signaling

					#9580
	Anti-BIN1	Western blot	1:500	Mouse Monoclonal	Santa Cruz sc-23918
	Anti-CASQ2	Western blot	1:500	Mouse Monoclonal	Santa Cruz sc-16693
Secondary	Goat anti-Mouse IgG Alexa Fluor 594	Immunofluorescence	1:200	Goat anti-Mouse IgG	Invitrogen A21145
	Goat anti-Rabbit IgG Alexa Fluor 488	Immunofluorescence	1:200	Goat anti-Rabbit IgG	Invitrogen A32731
	Chicken anti-Rabbit IgG Alexa Fluor 594	Immunofluorescence	1:200	Chicken anti-Rabbit IgG	Invitrogen A21442
	Chicken anti-Mouse IgG Alexa Fluor 488	Immunofluorescence	1:200	Chicken anti-Mouse IgG	Invitrogen A21200
	Goat anti-Rabbit IgG (H + L) IRDye 800CW	Western blot	1:20000	Goat anti-Rabbit IgG	LI-COR 926-32211
	Goat anti-Mouse IgG (H + L) IRDye 800CW	Western blot	1:20000	Goat anti-Mouse IgG	LI-COR 926-32210

Supplementary data Fig.1

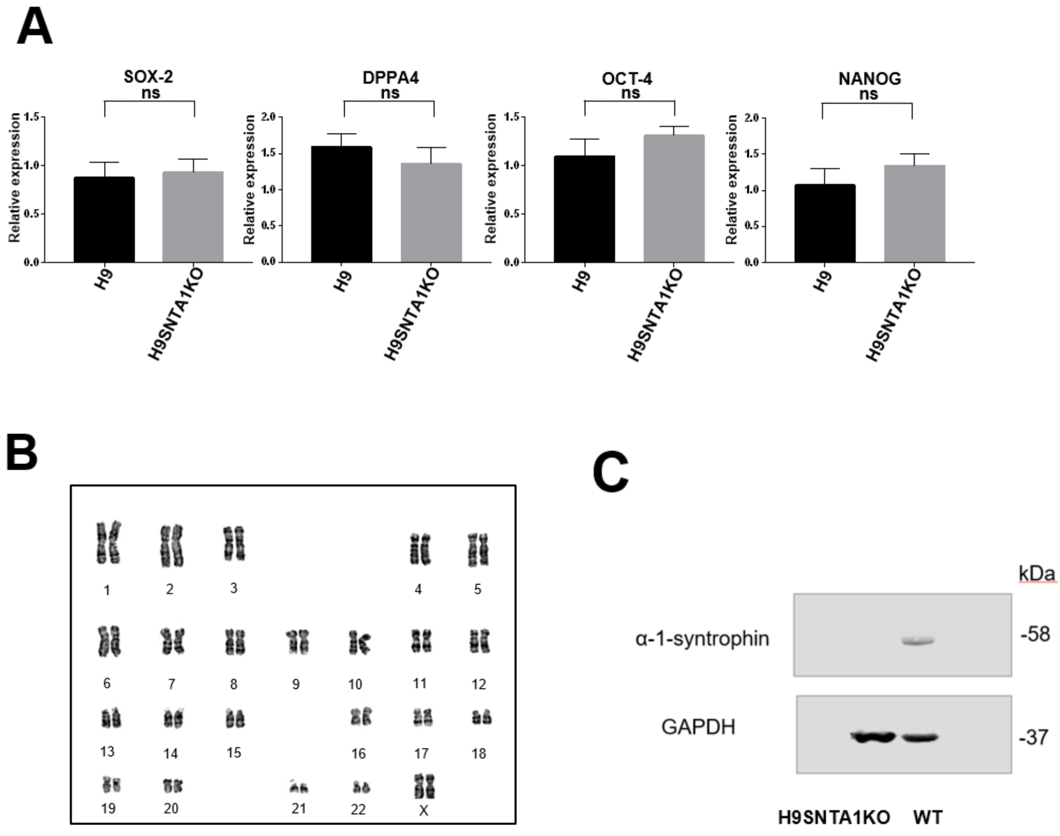

Supplementary data Fig.1 legend

A. Comparison of multipotent markers between H9 cells and H9SNTA1KO cells. The expression of pluripotent markers SOX2, DPPA4, OCT-4, and NANOG in H9SNTA1KO cells was similar to H9 cells using qRT-PCR analysis.

B. The karyotype analysis of H9SNTA1KO was normal (46, XX).

C. Western blots confirmed H9SNTA1KO to be actually deficient in SNTA1.

Supplementary data Fig.2

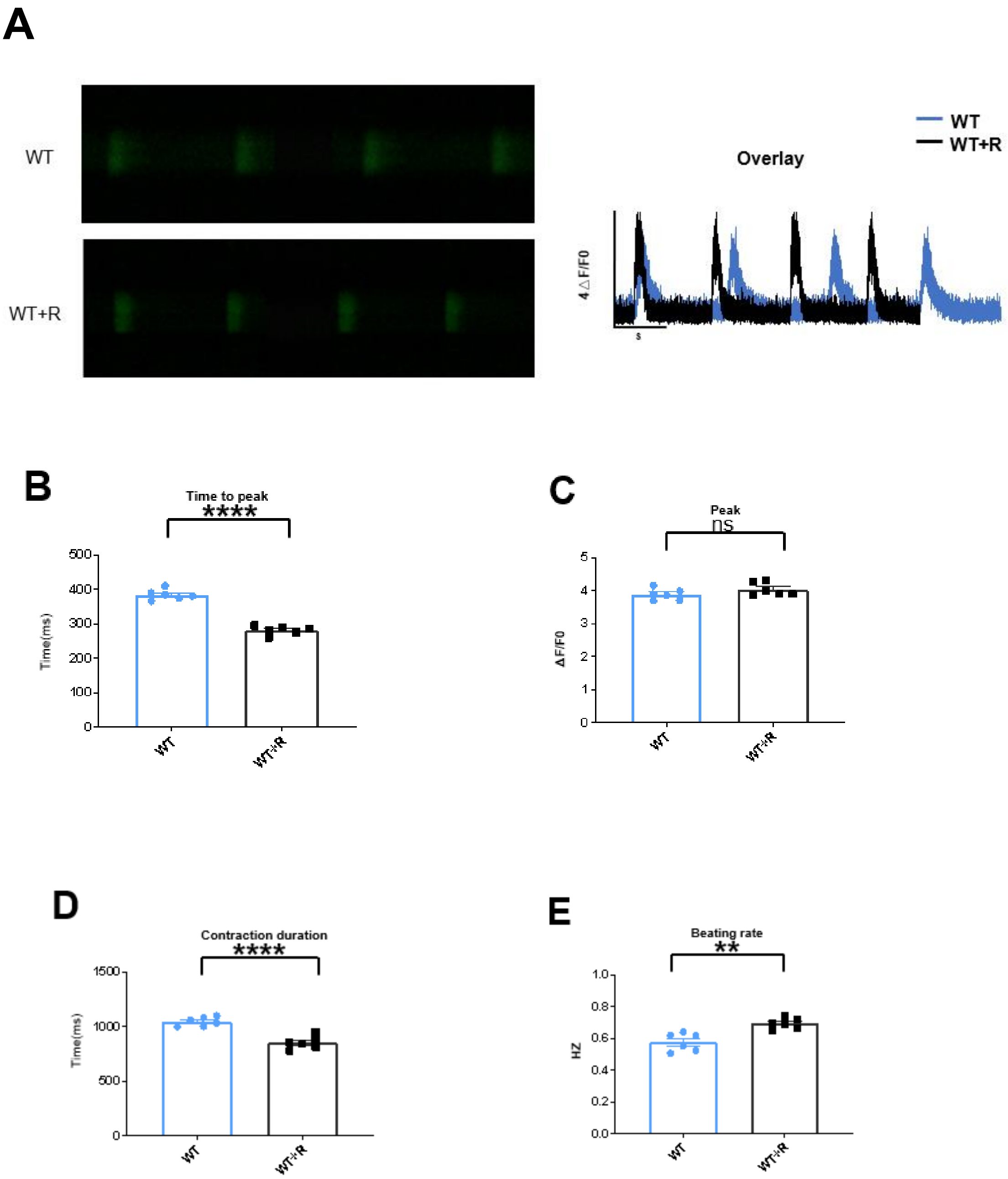

Supplementary data Fig.2 legend

A. The left panel: The representative line-scan image of WT and WT+R (WT cardiomyocytes treated with 10 μM ranolazine) stained with Fluo-4 AM.

The right panel: Calcium transient profile derived from line-scan image of the left panel.

B. to E. Quantification of time to peak, peak, contraction duration, and beating rate in WT and WT+R (WT cardiomyocytes treated with 10 μM ranolazine), n = 6. $^{**}P < 0.01$; $^{****}P < 0.0001$; ns; not significant, unpaired two-sided Student's t test.
